# Personalized neoantigen pulsed dendritic cell vaccine for advanced lung cancer

**DOI:** 10.1038/s41392-020-00448-5

**Published:** 2021-01-20

**Authors:** Zhenyu Ding, Qing Li, Rui Zhang, Li Xie, Yang Shu, Song Gao, Peipei Wang, Xiaoqing Su, Yun Qin, Yuelan Wang, Juemin Fang, Zhongzheng Zhu, Xuyang Xia, Guochao Wei, Hui Wang, Hong Qian, Xianling Guo, Zhibo Gao, Yu Wang, Yuquan Wei, Qing Xu, Heng Xu, Li Yang

**Affiliations:** 1 State Key Laboratory of Biotherapy and Cancer Center, West China Hospital, Sichuan University, and Collaborative Innovation Center for Biotherapy, Chengdu, 610041 China; 2 Department of Biotherapy, Cancer Center, West China Hospital, Sichuan University, Chengdu, China; 3 Department of Oncology, Cancer Center, Shanghai Tenth People’s Hospital, Tongji University, Shanghai, 200072 China; 4 Department of Radiology, West China Hospital, Sichuan University, Chengdu, Sichuan China; 5 YuceBio Technology Co., Ltd, Shenzhen, China

**Keywords:** Cancer, Vaccines

## Abstract

Neoantigens are considered to be ultimate target of tumor immunotherapy due to their high tumor specificity and immunogenicity. Dendritic cell (DCs) vaccines based on neoantigens have exciting effects in treatment of some malignant tumors and are a promising therapeutic modality. Lung cancer is a lethal disease with the highest morbidity and mortality rate in the world. Despite the rapid development of targeted therapy and immune checkpoint inhibitors for lung cancer in recent years, their efficacy is still unsatisfactory overall. Therefore, there is an urgent unmet clinical need for lung cancer treatment. Here, we attempted to treat lung cancer using a personalized neoantigen peptide-pulsed autologous DC vaccine and conducted a single-arm, 2 medical centers, pilot study initiated by the investigator (ChiCTR-ONC-16009100, NCT02956551). The patients enrolled were patients with heavily treated metastatic lung cancer. Candidate neoantigens were derived from whole-exome sequencing and RNA sequencing of fresh biopsy tissues as well as bioinformatics analysis. A total of 12 patients were enrolled in this study. A total of 85 vaccine treatments were administered with a median value of 5 doses/person (range: 3–14 doses/person). In total, 12–30 peptide-based neoantigens were selected for each patient. All treatment-related adverse events were grade 1–2 and there were no delays in dosing due to toxic effects. The objective effectiveness rate was 25%; the disease control rate was 75%; the median progression-free survival was 5.5 months and the median overall survival was 7.9 months. This study provides new evidence for neoantigen vaccine therapy and new therapeutic opportunities for lung cancer treatment.

## Introduction

Lung cancer is one of the malignant tumors with the fastest growth in morbidity and mortality that threatens human health and life.^1^ At present, surgery and definitive chemoradiotherapy remain the standard of care for early-stage lung cancer.^2^ For patients with advanced disease, targeted therapeutic drugs and immune checkpoint inhibitors (ICIs) have significantly improved the prognosis of some patients.^3–7^ However, those with advanced disease who failed in frontline therapies have limited therapeutic options and a dismal prognosis. The main goal in the management of recurrent advanced disease is, thus, to extend the survival, palliate symptoms, and improve the quality of life. Novel, effective and low toxicity therapy methods are urgently needed.

Several strategies, such as ICIs, adoptive cell therapy (ACT) and cancer vaccines, that harness the exquisite specificity of the immune system to eliminate tumors have emerged during the past decades.^8^ A growing body of evidence indicates that neoantigens, a kind of tumor antigen derived from tumor-specific somatic mutations, underlie the success of ICI therapy and ACT.^9–12^ In support of these findings, studies have shown that the adoptive transfer of selected tumor infiltrating lymphocytes (TILs) targeting neoantigens led to significant tumor regression.^13–15^ Therefore, increasing attention has been shifted to identifying neoantigens as antitumor targets.^16–18^

Neoantigen-based RNA or peptide vaccines have shown promising therapeutic effects in melanoma and glioblastoma.^19–22^ Dendritic cells (DCs) as vectors for antigen delivery are a major focus of cancer vaccines.^23,24^ Some studies reported that antigen-loaded DC vaccines induced stronger immune responses than vaccines composed of antigens and adjuvants.^25,26^ Therefore, neoantigen-based DC vaccines for cancer treatment seem to be promising and have been extensively investigated.^24^ To date, neoantigen-based DC vaccines have shown clinical success in melanoma and other solid tumors.^27,28^ Lung cancer has a high tumor mutation burden and a high level of tumor neoantigens.^29^ Neoantigen-based DC vaccine therapy should be a reasonable treatment option for these patients. However, currently, no prospective clinical trials focusing on lung cancer have been published.

In this study, we conducted a pilot study to investigate the safety and efficacy of a personalized neoantigen peptide-pulsed autologous DC vaccine (hence forth referred to as Neo-DCVac) in the treatment of advanced lung cancer patients. Preliminary results indicated that Neo-DCVac was feasible, safe, and effective. Neo-DCVac could elicit antigen-specific T-cell responses and induce antitumor immunity. We report the results of our study here.

## Results

### Patients and neoantigen identification

**Fig. 1:**
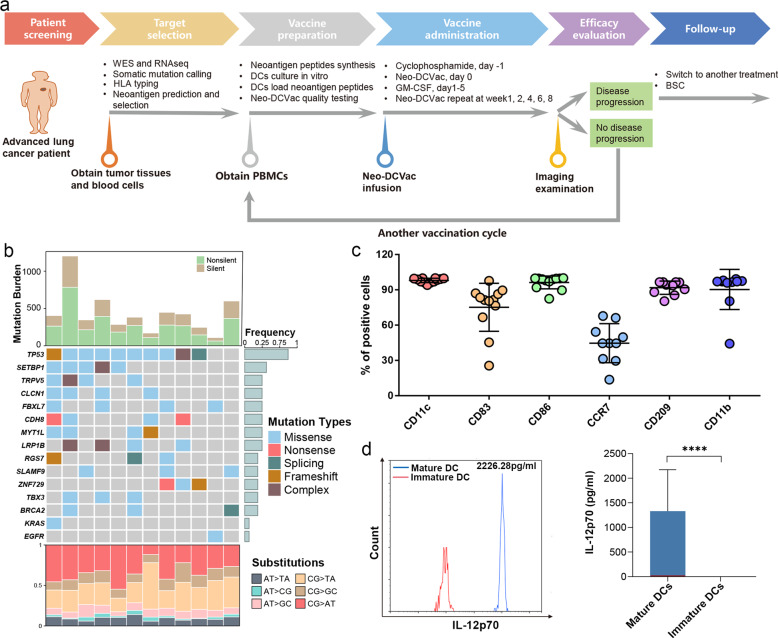
Diagram of vaccine workflow, overall mutational landscape, and analysis of DC vaccine products. **a** The treatment protocol of Neo-DCVac for patients with advanced lung cancer. BSC best support care. **b** The overall mutational landscape of the tumors from the 12 enrolled patients. Top, mutation burden; bottom, distribution of nucleotide changes; middle, mutated genes in the 12 lung cancer patients, arranged by the numbers of recurrences. **c** Percentage of output DCs expressing CD11c, CD83, CD86, CCR7, CD209, and CD11b in the vaccine products (*n* = 12). **d** Representative example of IL-12p70 secretion in immature and mature DCs (*n* = 12). All the data are shown as the mean ± s.e.m. (****P < 0.0001)

We conducted a single-arm, multicenter, pilot study to test the efficacy of Neo-DCVac in patients with relapsed advanced lung cancer. The Neo-DCVac formulation and the overall schedule of administration are shown in Fig. 1a. From November 2017 to September 2019, West China Hospital (*n* = 15) and the Tenth People’s Hospital of Tongji University (*n* = 3) recruited 18 patients with advanced lung cancer who had relapsed under standard multiline treatment. Except for patient 3 who had failed biopsies, tumor and blood samples were collected for high-throughput sequencing from 17 of 18 patients. After performing whole-exome sequencing (WES) on the DNA from both tumor and blood samples, a median of 312 (range, 80–808) somatic nonsynonymous mutations were identified in each patient (Supplementary Table 1, Supplementary Tables 3–14). Meanwhile, WES-based human leukocyte antigen (HLA) haplotypes were estimated and used for subsequent neoantigen predictions. Additionally, RNA sequencing (RNA-seq) was also performed for each tumor sample, not only confirming the expression status of the mutations but also detecting possible fusion-based neoantigens. After stepwise filtering criteria, 13–30 peptide-based neoantigens were selected for each patient (Supplementary Table 1). Five patients were excluded due to an insufficient number of actionable neoepitopes (patients 5 and 11), a loss of heterogeneity in HLA (patient 13) and death from rapid tumor progression (patients 9 and 10). For the remaining 12 patients, the patient demographics and baseline clinical characteristics are listed in Table 1. The overall mutational landscape is presented in Fig. 1b. The number of mutations in the *TP53* gene ranks at the top, while *EGFR* mutations were detected in only one patient.

**Table 1:** Baseline clinical characteristics of patients

Patient ID	Age	Sex	Pathology	Smoking	ECOG PS	Driver genes	Tumor stage	Distant metastases	Lines of prior therapy	Prior ICIs therapy
1	72	M	Adeno	N	0	WT	T4N2M1a (IV)	Lung	3	N
2	48	M	SqCC	Y	1	WT	T4N2M1c (IV)	Chest wall, bone	3	N
4	61	M	SqCC	Y	0	WT	T4N3M0 (IIIc)	NA	2	N
6	59	M	Adeno	Y	1	WT	T3N2M1b (IV)	Liver	3	N
7	73	F	Adeno	N	1	EGFR L858R	T4N1M1c (IV)	Brain	2	N
8	55	F	Adeno	N	1	EGFR exon19Del	T4N0M1c (IV)	Pleura, adrenal	4	N
12	55	M	SqCC	Y	1	WT	T4N0M1a (IV)	Lung	4	N
14	47	M	SCLC	Y	1	NA	T4N3M1c (IV, extensive)	Adrenal, Brain Subcutaneous, Bone	3	N
15	67	M	SCLC	Y	1	NA	T2N3M1c (IV, extensive)	Lung, Brain	4	Y
16	68	M	SqCC	Y	1	WT	T3N1M1a (IV)	Lung	7	Y
17	57	M	Adeno	Y	1	KRAS	T2aN0M1c (IV)	Bone, Adrenal, Lymph nodes, Pelvic	3	Y
18	69	M	SqCC	N	1	MET amplification	T2N2M1b (IV)	Brain	6	Y

*M* male, *F* female, *Adeno* adenocarcinoma, *SqCC* squamous cell carcinoma, *SCLC* small-cell lung cancer, *N* no, *Y* yes, *ECOG PS* Eastern Cooperative Oncology Group performance status, *WT* wild-type, *EGFR* epidermal growth factor receptor, *NA* not available, *ICIs* immune checkpoint inhibitors

### Feasibility of Neo-DCVac manufacturing

The peptides were synthesized according to the prediction algorithm. Following peptide synthesis, peripheral blood mononuclear cells (PBMCs) were collected by leukapheresis and cultured in the presence of human granulocyte-macrophage colony-stimulating factor (GM-CSF) and human interleukin-4 (IL-4) to induce differentiation into DCs. DCs were then pulsed overnight with neoantigen peptides to prepare the Neo-DCVac. Manufacturing of a Neo-DCVac was feasible in all patients. We performed a total of 17 events of leukapheresis for 12 patients, obtained a median of 2.55 × 10^9^ (range, 4.14 × 10^9^–1.50 × 10^9^) PBMCs and generated a median of 1.80 × 10^8^ (range, 0.70 × 10^8^–4.33 × 10^8^) DCs, showing that the median yield rate of DCs from PBMCs was 8.37% (range, 2.73–20.83%) (Supplementary Fig. 1a–c). A total of 85 doses of Neo-DCVac were generated with a median of five doses per patient (range, 3–14; Supplementary Fig. 1d). The median viable cell percentage of these Neo-DCVac products was 81% (range, 71–94%; Supplementary Fig. 1e), and the median number of viable cells per dose vaccine was 1.60 × 10^7^ (range, 0.65 × 10^7^–2.4 × 10^7^; Supplementary Fig. 1f). The average percentage of these Neo-DCVac products expressing the mature DC phenotypic markers CD11c and CD86 was >90%, and the average percentage of Neo-DCVac products expressing CD83 was >70% (Fig. 1c). The average percentages of antigen-presenting correlation phenotypic markers CD11b and CD209 were >90% (Fig. 1c). The average percentage of CCR7 was >40% (Fig. 1c). Compared with immature DCs, mature DCs showed significantly increased secretion of IL-12p70 (Fig. 1d). These DC vaccine products were negative for mycoplasma, bacteria or fungi and contained an endotoxin (<5 EU/ml). The median time from biopsy to first vaccination was 2.8 months (range, 2.1–3.5 months).

### Safety of Neo-DCVac administration

Patients were pretreated with cyclophosphamide at a dose of 250 mg/m^2^ 1 day before injection of Neo-DCVac. The prepared Neo-DCVac was vaccinated subcutaneously at both axillary and inguen regions bilaterally at day 0. GM-CSF was administered at a dose of 0.075 mg during the following 5 days (days 1–5). Safety and tolerability were assessed in the 12 patients who received at least one dose of Neo-DCVac. Neo-DCVac treatments were well tolerated, and all adverse events were limited to grade 1 or 2 (Table 2). All patients experienced grade 1 skin injection-site reactions. Patient 4 developed transient grade 1 neutropenia after receiving Neo-DCVac treatment, which was relieved without any treatment. Patients 12 developed a grade 2 itchy rash throughout his trunk and extremities (Table 2). No other specific treatment was administered, and his rash waned after vaccination ceased. Among the five patients who used ICIs during the vaccine immunization period (four patients continued previous ICIs treatment: patient 15 and patient 16 continued to receive nivolumab, patient 17 continued with pembrolizumab and ipilimumab, patient 18 proceeded with pembrolizumab, and 1 patient received nivolumab in combination with vaccine treatment), Neo-DCVac did not increase the risk of immune-related adverse events related to ICIs. No toxicity was dose-limiting or resulted in dose delay or treatment discontinuation.

**Table 2:** Treatment-related adverse events in all treated patients^a^

	All treated patients (*n* = 12)	
	Grade 1–2	Grade 3–4
Constitutional		
Injection-site reaction	12 (100%)	0
Flu-like symptoms	0	0
Fever	0	0
Fatigue	0	0
Chills	0	0
Dizziness	0	0
Gastrointestinal		
Nausea	0	0
Constipation	0	0
Vomiting	0	0
Diarrhea	0	0
Dry mouth	0	0
Respiratory		
Cough	0	0
Dyspnea	0	0
Laboratory		
Anemia	0	0
Neutropenia	1 (8.3%)	0
Other		
Rash	1 (8.3%)	0
Headache	0	0

^a^Including all patients who received at least one dose of a trial treatment

### Overall effectivity of Neo-DCVac therapy

**Fig. 2:**
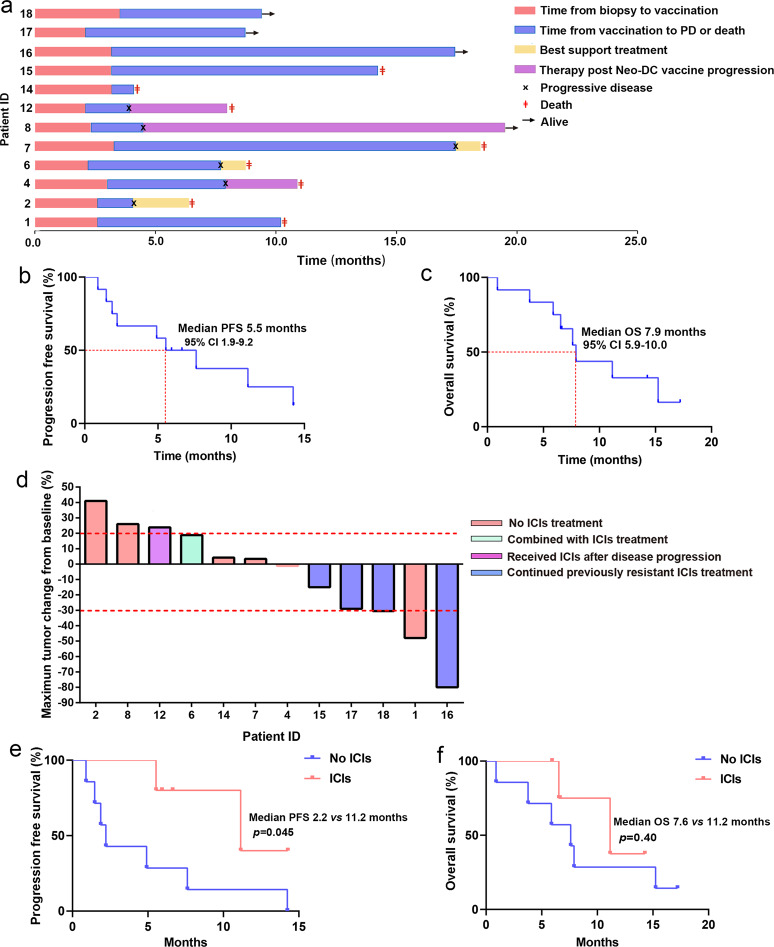
Presupposition analysis of the clinical activity of Neo-DCVac in the treatment of advanced lung cancer. **a** Clinical event timeline for the 12 patients who received Neo-DCVac treatment, from biopsy until the time of death due to progressive disease (PD) or last follow-up time. **b** Progression-free survival (PFS) of patients treated with Neo-DCVac. **c** Overall survival (OS) of patients treated with Neo-DCVac. **d** Best observed response for 12 patients given Neo-DCVac with or without ICI treatment. **e** PFS of patients treated with Neo-DCVac or Neo-DCVac and ICIs. **f** OS of patients treated with Neo-DCVac or Neo-DCVac and ICIs

Each vaccination cycle consisted of 5 repeats at weeks 1, 2, 4, 6, and 8. At the end of each vaccination cycle, a thorough radiographic examination was performed, and the efficacy was evaluated per Response Evaluation Criteria in Solid Tumors (RECIST), version 1.1 criteria. If the tumor was under control, the patient continued to receive vaccine treatment; otherwise, the patient was switched to other therapies as the tumor progressed. At the time of the analysis, eight patients had died, and 4 of them were still receiving treatment (Fig. 2a). After disease progression, patients 2, 6, and 7 received the best supportive treatment (BST), patient 4 received palliative radiotherapy for lung tumor lesions, patient 8 received chemotherapy and osimertinib, and patient 12 received PD-1 inhibitor (nivolumab) treatment (Fig. 2a). Of the six patients with disease control over 6 months, patient 1 was a male with metastatic lung adenocarcinoma; after failure of three lines of therapy, he subsequently underwent personalized Neo-DCVac treatment, and his tumor achieved a partial response (PR) for 7.6 months. Patient 7 was a female with adenocarcinoma harboring an EGFR exon 21 mutation (L858R) and had extensive brain metastases. New brain metastases continued to emerge while receiving the tyrosine kinase inhibitor erlotinib. After vaccination, her brain metastases were controlled for over 1 year. Patient 15 was a male with extensive-stage small-cell lung cancer (SCLC). After the failure of previous multiline therapy, Neo-DCVac made the patient’s disease stable for nearly 1 year. Patients 16 and 18 were 2 male patients with intensively treated lung squamous cell carcinoma (7 and 6 lines of previous treatment, respectively; both received ICIs). After combined treatment with Neo-DCVac and ICIs, their tumor target lesions were reduced by 80% and 30.2%, respectively. Patient 17 was a male with extensive-stage lung adenocarcinoma. After the failure of previous multiline therapy including PD-1 inhibitors, patient 17 achieved a response of stable disease (SD) with Neo-DCVac. The median duration of follow-up in this cohort of patients was 7.1 months (range, 0.9–17.2). The median progression-free survival (PFS) was 5.5 months (95% confidence interval (CI, 1.9–9.2), and the median overall survival (OS) was 7.9 months (95% CI, 5.9–10.0) (Fig. 2b, c). Three (25%) of 12 patients achieved an objective response (Fig. 2d, Supplementary Table 2). All of the responses were PR, with no complete responses (CRs) recorded. Six (50%) of 12 patients experienced a decrease in the size of their target lesions, nine (75%) of 12 patients achieved disease control, and progressive disease (PD) was recorded in three patients (25%) (Fig. 2d, Supplementary Table 2).

### Synergistic therapeutic effects of Neo-DCVac with ICIs

In total, four patients who received ICIs treatment were recruited in our trial, exhibiting either primary no response to or relapse from such therapy. After combining ICIs treatment with Neo-DCVac, all these patients achieved disease control (2 PRs and 2 SDs), and tumor sizes were reduced by up to 80% (Fig. 2d, Supplementary Table 2). According to the clinical report, three of these patients are still alive, and only one patient with SCLC died; the OS is much worse for SCLC than for non-small-cell lung cancer (NSCLC). The clinical experience of patient 6 who received combination therapy of PD-1 inhibitor (nivolumab) and Neo-DCVac should be noted. In particular, although the post-treatment PBMCs showed a stronger response to three neoantigen peptides, the tumor in the upper left lung of patient 6 increased, and the live metastasis after immunization decreased. Interestingly, when nivolumab was later used in combination with the vaccine, his swollen tumor became cavitated (Supplementary Fig. 2). Collectively, the combination therapy of ICIs and Neo-DCVac had a longer PFS and a better OS trend (patient 12 received a post-PD-1 inhibitor after failure of Neo-DCVac and was not included in the analysis) (Fig. 2e, f), indicating a potential synergistic therapeutic effect of these two treatment strategies. However, the sample size was too small, preventing us from drawing reliable conclusions.

### Case analysis of Neo-DCVac treatment

**Fig. 3:**
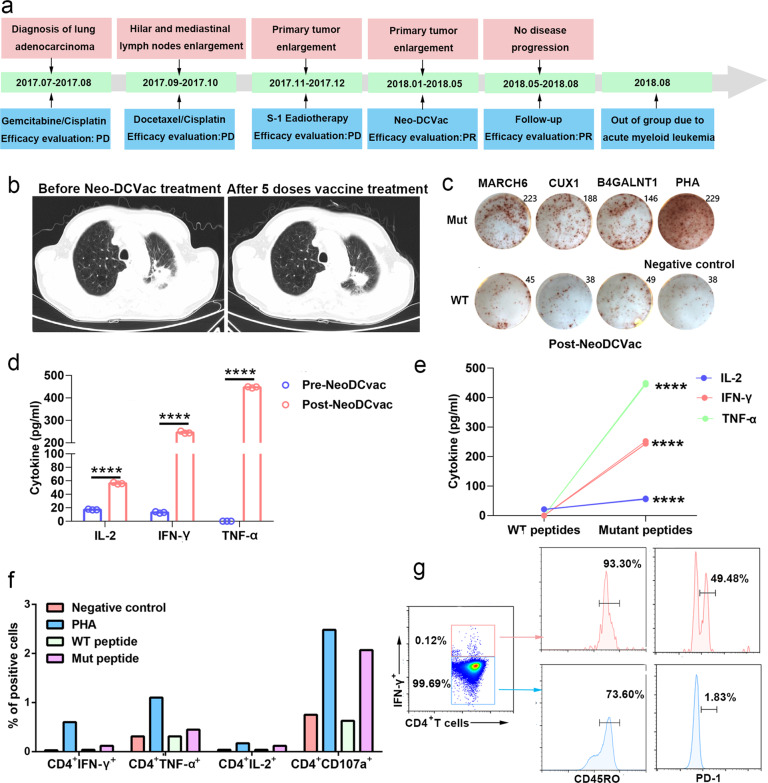
Clinical and immune responses to personalized Neo-DCVac in patient 1 with metastatic lung adenocarcinoma. **a** Clinical course of the disease and previous treatments of patient 1. The patient failed three lines of chemotherapy and radiotherapy but remained without any signs of disease progression for 7.6 months following Neo-DCVac administration. **b** Computed tomography (CT) scans were performed before and after five doses of personalized Neo-DCVac immunotherapy, and representative images are shown. **c** Autologous PBMCs were stimulated with 13 candidate mutant peptides for 10 days, after which IFN-γ ELISpot assays were performed to assess the T-cell-specific antigen response. One percent phytohemagglutinin (PHA) and no peptide stimulation represent the positive and negative controls, respectively. The IFN-γ ELISpot picture of fold changes of mutant peptides/WT peptides >2 is shown. **d** Before and after Neo-DCVac treatment, the PBMCs were stimulated with neoantigen peptides overnight, and the concentrations of IL-2, IFN-γ, and TNF-α in the culture supernatants were measured by cytometric bead array (CBA). Data are representative of results from three independent experiments (*n* = 3). This data are shown as the mean ± s.e.m. **e** After Neo-DCVac treatment, the concentrations of IL-2, IFN-γ, and TNF-α in the culture supernatant were measured by CBA. The data represent the results of three independent experiments. **f** Analysis of ex vivo T-cell responses to neoantigen peptides after overnight exposure to neoepitopes and corresponding WT peptides using intracellular cytokine staining followed by flow cytometry. **g** Ex vivo intracellular cytokine staining of PBMCs after neoepitope stimulation. PBMCs were pre-gated on CD3^+^ and CD4^+^ T cells. Cytokine-producing neoantigen-reactive cells express CD45RO and PD-1, demonstrating an antigen-experienced memory T-cell phenotype (****P < 0.0001)

Patient 1 was a 72-year-old man with metastatic lung adenocarcinoma who was subsequently enrolled and received individualized Neo-DCVac treatment after failure of three lines of chemotherapy and radiotherapy (Fig. 3a). Information on the neoantigens used by this patient is shown in Supplementary Table 3. Computed tomography (CT) scans performed after a round of Neo-DCVac revealed PR of the primary lung tumor (Fig. 3b), and post-treatment PBMCs showed stronger responses against mutated MARCH6, CUX1, and B4GALNT1 neoepitopes than those against the corresponding wild-type (WT) peptides (Fig. 3c). Compared with the baseline, post-Neo-DCVac PBMCs showed increased secretion of IL-2, interferon-γ (IFN-γ), and tumor necrosis factor-α (TNF-α) as measured by a cytometric bead array (CBA) after being stimulated by the mutated MARCH6, CUX1, and B4GALNT1 peptides (Fig. 3d). Additionally, compared with the corresponding WT peptide stimulation, the mutant peptide-stimulated PBMCs, which were obtained after vaccination, showed significantly increased cytokine release (Fig. 3e). In addition, after stimulation with mutant peptides, CD3^+^CD4^−^ T cells and CD3^+^CD4^−^ T cells from post-Neo-DCVac PBMCs secreted IFN-γ, IL-2, TNF, and CD107a based on direct ex vivo intracellular cytokine staining, thus demonstrating that these T cells recognized the mutant peptides after vaccination (Fig. 3f, Supplementary Fig. 3a–b). The IFN-γ-producing CD4^+^ T cells were enriched for analysis, and both CD45RO and PD-1 were highly expressed, which was consistent with the generation of antigen-experienced memory T cells following vaccination (Fig. 3g, Supplementary Fig. 3c–d).

**Fig. 4:**
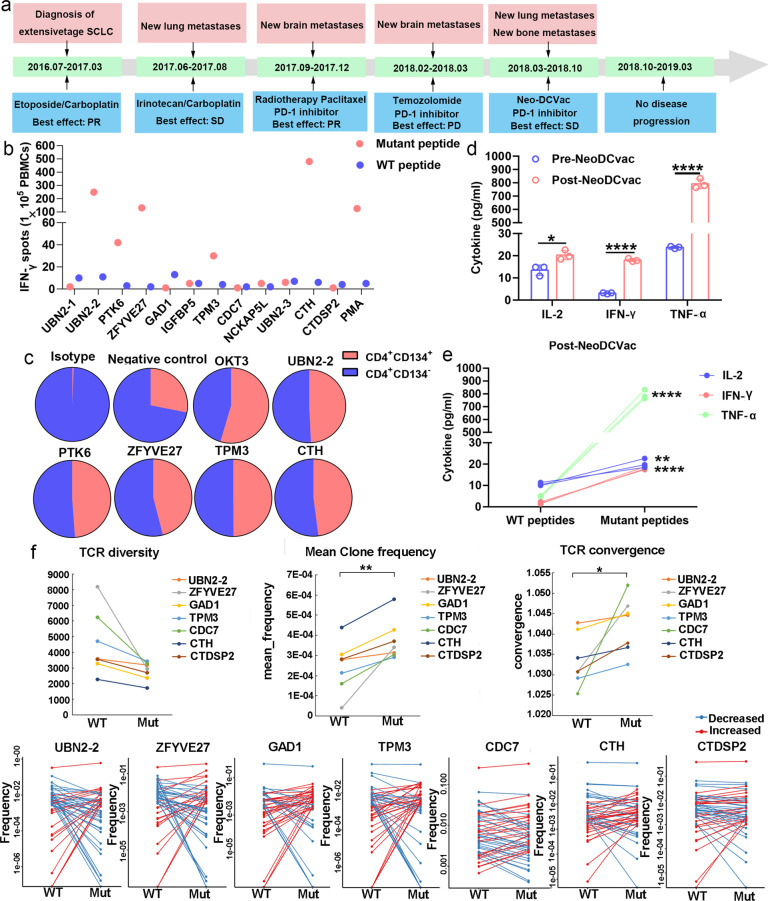
Immune responses to personalized Neo-DCVac in patient 15 with extensive-stage SCLC. **a** Clinical course of the disease and previous treatments of patient 15. The patient failed four lines of chemotherapy and radiotherapy but remained without any sign of disease progression for 11.2 months following Neo-DCVac administration. **b** Autologous PBMCs were stimulated with 12 candidate mutant peptides and corresponding WT peptides for 10 days, after which IFN-γ ELISpot assays were performed to assess the T-cell-specific antigen response. One percent phytohemagglutinin (PHA) and no peptide stimulation represent the positive and negative controls, respectively. **c** Autologous PBMCs were stimulated with candidate mutant peptides every 3 days in the presence of IL-2, and on day 10, T-cell responses to each antigen were measured by flow cytometric analysis for CD134 upregulation on CD4^+^ T cells (gated on CD3). The no peptide stimulation was used as a negative control, and OKT3 stimulation was used as a positive control. **d** Before and after Neo-DCVac treatment, the PBMCs were stimulated with neoantigen peptides overnight, and the concentrations of IL-2, IFN-γ, and TNF-α in culture supernatants were measured by cytometric bead array (CBA). Data are representative of results from three independent experiments (*n* = 3). This data are shown as the mean ± s.e.m. **e** Post-Neo-DCVac treatment, the concentrations of IL-2, IFN-γ, and TNF-α in culture supernatants were measured by CBA after exposure to neoepitopes and corresponding WT peptides. Data are representative of results from three independent experiments (*n* = 3). **f** T-cell receptor (TCR) diversity, mean clone frequency, TCR convergence and the top ranked TCR clones in terms of absolute frequency change were detected after treatment with the WT peptide or mutant peptide. (*P < 0.05, **P < 0.01, ****P < 0.0001)

Patient 15 was a male with extensive-stage SCLC. Information on the neoantigens used by this patient is shown in Supplementary Table 4. After the failure of previous multiline therapy, he received Neo-DCVac treatment (Fig. 4a). Following 5 doses of Neo-DCVac, he achieved SD with a 15% maximum reduction in target lesions compared to baseline. Eventually, the Neo-DCVac stabilized the patient’s condition for nearly a year. In the detection of specific responses to neoantigens, we found that the PBMCs obtained after vaccination had significantly stronger responses to the five mutant peptides than the corresponding WT peptides (Fig. 4b); the CD8^+^ T cells obtained after vaccination had significantly stronger responses to the three mutant peptides than the corresponding WT peptides; and the CD4^+^ T cells obtained after vaccination had significantly stronger responses to the six mutant peptides than the corresponding WT peptides (Supplementary Fig. 4a–4b). Moreover, using candidate mutant peptides to stimulate PBMCs obtained after vaccination, we found that the proportion of CD134^+^ cells in CD4^+^ T cells was significantly increased (Fig. 4c). In terms of cytokine secretion, the secretion of antigen-specific IL-2, IFN-γ, and TNF-α was significantly increased after vaccination (Fig. 4d, e). These data indicate that Neo-DCVac can successfully activate some T cells and kill tumor cells, suggesting that Neo-DCVac can be used for tumor treatment. In addition, significantly decreased T-cell receptor (TCR) clone diversity, increased mean frequency of clones and increased TCR convergence were observed after mutant peptide incubation, indicating the impact of neoantigens on TCRs. Particularly, after ranking the top TCR clones in terms of absolute frequency changes, more than a 1000-fold increased frequency was identified for several TCR clones in samples from mutated peptides compared to those from WT peptides, suggesting that the T-cell clones with these specific TCRs may directly contribute to the immune response (Fig. 4f).

**Fig. 5:**
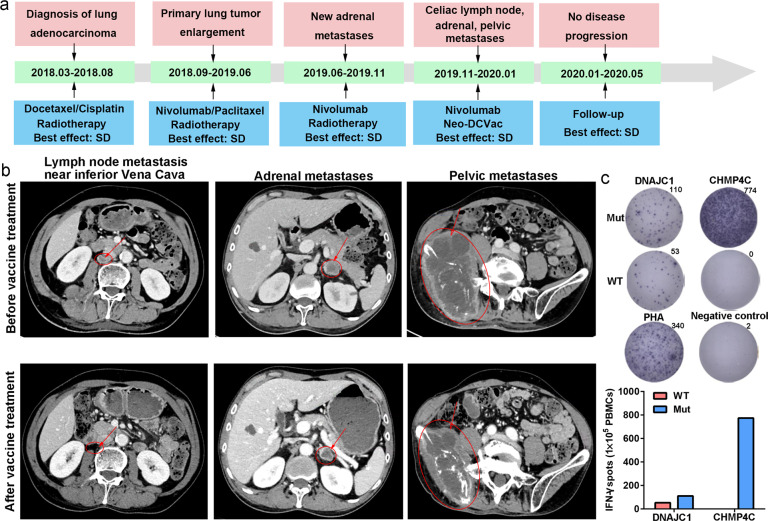
Clinical and immune responses to personalized Neo-DCVac in patient 17. **a** Clinical course of the disease and previous treatments of patient 17. The patient failed three lines of chemotherapy, radiotherapy and PD-1 inhibitor, and his tumor target lesions were reduced by 29% following Neo-DCVac administration. **b** Computed tomography (CT) scans were performed before and after personalized Neo-DCVac immunotherapy, and representative images are shown. **c** Autologous PBMCs were stimulated with 15 candidate mutant peptides for 10 days, after which IFN-γ ELISpot assays were performed to assess the T-cell-specific antigen response

Patient 17 was a male with extensive metastatic lung adenocarcinoma. The main metastases were located in the bone, pelvis, adrenal glands, and inferior vena cava lymph node. Information on the neoantigens used by this patient is shown in Supplementary Table 5. After the failure of three lines of therapies, including a PD-1 inhibitor (nivolumab), he received Neo-DCVac treatment (Fig. 5a). After five doses of Neo-DCVac, the metastatic lymph node almost disappeared, and the adrenal gland and pelvic metastatic lesions were shrinking (Fig. 5b). Overall, his tumor target lesions were reduced by 29%. In the detection of specific responses to neoantigens, we found that the PBMCs obtained after vaccination had significantly stronger responses to the two mutant peptides than to the corresponding WT peptides (Fig. 5c). These results indicate that the vaccine played a therapeutic role in the tumor treatment of this patient.

## Discussion

For patients with recurrent advanced disease, it is very important to explore a treatment method that can exert antitumor effects without increasing toxic and side effects. In this study, we show that Neo-DCVac is safe and tolerable and can be conveniently administered for patients with advanced lung cancer. All of the adverse events were low-grade and transient. More importantly, Neo-DCVac did not increase the severity of ICI immunotherapy or induce additional adverse events associated with ICI immunotherapy. In our study, combination therapy of Neo-DCVac and ICIs did not lead to any treatment discontinuation. Furthermore, there were no dose-limiting or grade 4 or 5 adverse events recorded with Neo-DCVac or with combination therapy of Neo-DCVac and a PD-1 inhibitor.

Although none of the enrolled patients achieve CR, this pilot study demonstrated a 25% objective response rate and 75% disease control rate (DCR) in patients with pretreated advanced lung cancer who were treated with a personalized Neo-DCVac. These results were obtained despite the poor baseline prognostic factors of our study population. In addition, an encouraging duration of response was observed in some patients.

Recently, some papers reported the successful induction of responses to tumor neoepitopes after vaccination using a neoantigen vaccine or whole-tumor lysate vaccine.^19–22,27,28,30^ We analyzed 10 evaluable patients in whom we could obtain PBMCs after immunization with Neo-DCVac. In all these patients, we documented PBMC responses against the predicted neoantigen peptides. Of the six patients with more than 8 T-cell response neoantigens, one patients had an objective responses and five patients achieved disease control. However, in the four patients with less than 8 T-cell response neoantigens, 50% of them experienced disease progression (Supplementary Table 2). We demonstrated that after immunization with Neo-DCVac, post immunization PBMCs recognized some predicted neoantigen peptides, and the number of neoantigens recognized by post immunization PBMCs seem to be related to clinical efficacy. However, for the relationship between the number of immunogenic neoantigens and clinical efficacy to be analyzed more accurately, the number of patients needs to be further increased.

Here, the accuracy of the in silico prediction of neoantigens is not high enough. Therefore, we picked at least 15 candidate peptides for each patient. The filtering criteria were described in the methods section. After excluding the candidates that were not expressed and had a low tumor variant allele frequency, we ranked the remaining candidates based on MHC affinity scores (IC50) and WT/mut ratio (Supplementary Tables 3–14). We could evaluate T-cell responses after treatment with these candidate peptides and estimated the responses with an ELISpot assay. After analysis, we did not determine the relationship between HLA affinity and T-cell responses, which have been due to the limitations of bioinformatics screening methods and other factors (e.g., protein levels of the neoantigen). In future research, we will further try to improve the screening methods for neoantigens and use whole-tumor lysates to prepare DC vaccines for experiments. In addition, we have added data on the percentage of neoantigens that can stimulate the response of antigen-specific CD4^+^ T cells and CD8^+^ T cells in the supplementary materials (Supplementary Table 15). However, in this study, we were temporarily unable to analyze whether the response range of CD8^+^ T cells and CD4^+^ T cells was related to disease response, because antigen-specific CD4^+^ T-cell and CD8^+^ T-cell responses were analyzed in only six patients.

In theory, the combination of a DC vaccine and ICIs can enhance the antitumor activity of T cells. PD-1 inhibitors block the binding of PD-1 to PD-L1; anti-CTLA4 monoclonal antibodies block the binding of CTLA4 to CD80 and CD86, and mature DCs highly express CD80 and CD86. Then, more CD80 and CD86 can bind to CD28, thus enhancing the antitumor ability of T cells. In mouse models, the combination of an anti-CTLA4 monoclonal antibody and a DC vaccine demonstrated enhanced antitumor activity.^31^ In a phase I clinical trial of 16 patients with melanoma treated with the combination of MART-1 peptide-pulsed DCs and tremelimumab, a higher rate of durable objective tumor responses was observed than what was expected from each agent alone.^32^ In our study, patient 6 had an enlarged tumor in the upper left lung after Neo-DCVac immunization (information on the neoantigens used by this patient is shown in Supplementary Table 6). However, his enlarged tumor became cavitated when a PD-1 inhibitor (nivolumab) was combined with Neo-DCVac (Supplementary Fig. 2). Furthermore, we report that Neo-DCVac can reinduce objective responses to ICI immunotherapy after treatment relapse or failure. The reinduction of response coincided directly with initiation of Neo-DCVac after progression in patients with relapsed disease treated with uninterrupted nivolumab. Moreover, disease control was asserted in all four patients who had relapsed disease after previous anti-PD-1 immunotherapy. Neo-DCVac is able to safely reinduce objective responses to immunotherapy in patients whose disease had relapsed after previous single-agent PD-1 monoclonal antibody treatment, suggesting that Neo-DCVac has important implications in solid tumor oncology beyond NSCLC.

Overall, our findings from this pilot study have proven that Neo-DCVac is feasible, safe, and capable of eliciting specific T-cell immunity and therapeutic benefit. To the best of our knowledge, we provide the first findings for the activity of a neoantigen-based DC vaccine treatment in patients with advanced NSCLC. Perhaps of greatest importance, Neo-DCVac could have implications in a wide range of cancers.

## Materials and methods

### Study oversight

The ethical committee of West China Hospital reviewed and approved the study protocol (2016–27). The study was performed in accordance with the Declaration of Helsinki and was registered at Chinese Clinical Trial Registry and Clinical Trials.gov (ChiCTR-ONC-16009100 and NCT02956551, respectively). This prospective study was conducted in 2 medical centers (West China Hospital and Tenth People’s Hospital of Tongji University, China), and patients were required to provide written informed consent prior to entering the study.

All the authors attest that the study was conducted in accordance with the protocol and all its amendments. All the authors had access to the data used for the writing of the manuscript and vouch for the accuracy and completeness of the data and analyses.

### Patients

Patients with histologically or cytologically confirmed lung cancer, including squamous cell carcinoma, adenocarcinoma, neuroendocrine tumor, and SCLC, who were aged between 18 and 75 years with at least one measurable disease according to the RECIST, version 1.1 were enrolled. Patient must have underwent standard of care therapies and have progressed diseases. The life expectance should be at least 3 months. Further entry criteria included an Eastern Cooperative Oncology Group (ECOG) performance status of 0 or 1, as well as adequate bone marrow, renal, and hepatic functions. To be eligible, patients must have an adequate wash-out period (for chemotherapy, >4 months, and for targeted therapy, >2 weeks). Patients who had progressed after immunotherapy with ICIs were eligible and had no requirements for the wash-out period.

Patients who were considered ineligible were those with a history of a second cancer. Other criteria of ineligibility included unstable systemic comorbidities, such as active gastric ulcer, grade 3 hypertension, unstable angina pectoris, congestive heart failure, active viral hepatitis, uncontrolled systemic infection, significant loss of body weight (>10% during the last 6 weeks), and other conditions of persistent medication of corticosteroids. Patients with tumors extremely difficult to biopsy were not included.

### Neoantigen identification

#### Sample collection and DNA/RNA preparation

Tumor tissues were obtained by fiber bronchoscopy biopsy (patient 4), supraclavicular metastatic lymph node resection (patient 14), fine needle aspiration of metastatic lumbar lesion (patient 17) and fine needle aspiration of lung tumor lesions (patients 1, 2, 5, 6, 7, 8, 9, 10, 11, 12, 13, 15, 16, and 18), and stored with 1 ml of RNA later (Thermo Fisher, #AM7020). Blood was collected and mixed with an EDTA-based anticoagulant. DNA and RNA were extracted from the same tumor tissue with the All Prep DNA/RNA Mini Kit (Qiagen), and DNA from EDTA-anti coagulated peripheral blood was extracted with the QIAamp DNA Blood Mini Kit (Qiagen). Quality of DNA/RNA was evaluated by 2100 Bioanalyzer (Agilent). Trio samples (two DNA samples from tumor and blood, and one RNA sample from only the tumor) for each patient were prepared for the subsequent sequencing process.

#### High-throughput sequencing

WES and RNA-seq for DNA/RNA trio samples from 17 patients were conducted by the Yuce Biotechnology Co., Ltd. Briefly, whole-exome capture was performed using an Agilent Sure Select Human All Exon 44 Mb version 2.0 bait set (Agilent Technologies), while RNA-seq libraries were prepared using Illumina’s TruSeq RNA Access Library Prep Kit according the manufacture instruction. The standard protocols from Illumina were followed to construct a sequencing library for WES and RNA-seq as previously described,^33^ and submitted to the Illumina HiSeq ×10 platform to generate sequencing data with 150 bp paired-end reads in the FASTQ format. Cleaned reads for DNA were aligned to the GRCh37/hg19 human reference genome using Burrows–Wheeler Aligner (BWA-MEM, v.0.7.12).^34^ Duplicate reads were removed, and InDel realignment and base quality score recalibration were performed with Genome Analysis Toolkit (GATK, v.4.0.10.1) according to GATK best practices. Clean reads for RNA were aligned to the GRCh37/hg19 human reference genome using STAR software (version.2.7.1a). GATK (v.4.0.10.1) was used to remove duplicate reads and split “N” cigar reads (i.e., splice junction reads) in the aligned reads.

#### Somatic mutation calling and gene expression

Somatic mutations were determined with aligned DNA sequencing data by Mutect2 in the GATK bundle in matched tumor and normal samples. A mutation in the tumor was identified as a candidate somatic mutation only when (i) >10 reads covered the mutation in both the tumor and normal samples; (ii) distinct paired reads contained the mutation in the tumor sample; and (iii) >10% reads covered the mutation in the tumor sample. Candidate somatic mutations were annotated with SnpEff^35^ and SnpSift (v.4.3).^36^ Subsequently, candidate somatic mutations were further filtered based on annotation, where retaining mutations should (i) occur with <0.001 frequency in the East Asian population according to genome aggregation database (gnomAD, v.2.1.1);^37^ (ii) be located in the coding regions; (iii) be nonsynonymous single nucleotide variations (SNVs) or in-frame InDels. Furthermore, amanual visual inspection with Integrative Genomics Viewer (IGV, v2.4.15)^38^ was used to further remove artificial changes. Somatic copy number alterations were assessed by CNVkit (v.0.9.2).^39^ Purities and ploidies of tumor samples were inferred with the ABSOLUTE algorithm (version 1.1).^40^ Prevalence of mutations were inferred with PyClone (v.0.13.0).^41^ Mutations with Cancer Cell Fraction (CCF) > 0.8 were defined as clonal mutations, and the others were defined as subclonal mutations. Genes expression values were quantified with feature Counts (v1.5.3)^42^ in Subread bundle according to the Gencode (v.19) annotation by using the aligned RNA-seq data. Haplotype Caller in the GATK bundle (v.4.0.10.1) was employed to obtain the mutated allelic frequency of somatic mutations in mRNA.

#### HLA typing

HLA haplotypes were predicted in silico using Polysolver (v.4)^43^ and HLAminer (v.1.4),^44^ where mapped reads from tumor DNA exome sequencing data were used. Loss of heterozygosity of HLA was estimated by comparing the HLA typing in tumor DNA with that in blood-derived germline DNA.

#### Neoantigen filtering

HLA binding affinity was estimated via the Immune Epitope Database and Analysis Resource (IEDB)-recommended mode of the IEDB T-cell prediction tools (version 2.5)^45^ using all variant-containing 8–14-mers for major histocompatibility complex (MHC) class I molecules or 15-mers for MHC class II molecules. Mutation-containing epitopes binding to HLAs with <500 nM affinity were defined as candidates for the subsequent filtering. The predicted neoepitopes were chosen for inclusion based on a predefined set of criteria in the following rank order: (i) strong binders with IC_50_ < 50 nM or % Rank < 0.5; (ii) higher affinity of mutated peptides than of matched WT peptides; (iii) mutations with higher tumor variant allele fraction (VAF); (iv) presentation of at least >5 reads cover mutated allele in RNA-seq results; and (v) mutations in oncogenes were given a higher priority within each ranked group; otherwise epitopes were ranked by predicted mutated peptide affinity. Additionally, a variety of possible biochemical properties (hydrophobicity or presence of multiple cysteines), which may affect the synthesizability or solubility of the long peptide were considered. A total of 13–30 predicted peptides for each patient were used for peptide synthesis.

### Vaccine preparation

#### Peptide synthesis

The peptides were 5–15 amino acids in length; peptides were synthesized by standard solid-phase synthetic peptide chemistry and purified using reversed-phase high-performance liquid chromatography (HPLC) (Shanghai Science Peptide Biological Technology Co., Ltd.). The type of HPLC used was LC-20AT (Shimadzu, Japan). The experimental conditions were Pump A: 0.1% trifluoroacetic in 100% water; Pump B: 0.1% trifluoroacetic in 100% acetonitrile; total flow: 1 ml/min; wavelength: 214 nm; analytial column type: SHIMADZU Inertsil ODS-SP (4.6 × 250 mm × 5 um).

#### DC generation

PBMCs were isolated from patients’ peripheral blood by COM.TEC (Fresenius Kabi, Germany) and then transferred to a good manufacturing practice (GMP)-compliant lab for in vitro processing and expansion. Monocyte-derived DCs were generated by plate adherence of PBMCs as described previously.^27,46^ Briefly, 5–10 × 10^6^/ml elutriated PBMCs were inoculated into T175 flasks containing AIM-V medium (Gibco, USA) and incubated for 3 hat 37 °C and 5% CO_2_. Then, the suspended cells were collected and frozen in liquid nitrogen. The adherent cells were washed and cultured in AIM-V medium containing 1% autologous serum, clinical grade human GM-CSF (1000 IU/ml; Hainan Unipul Pharmaceutical Co., Ltd. China), and animal-free research grade IL-4 (500 IU/ml; PrimeGene, China). After 5 days, the neoantigen peptides (50 µg/ml) were added to the immature DCs. Following 20–24 h peptide loading, DCs were matured with TNF-α (10 ng/ml; PrimeGene, China), IL-1β (10 ng/ml; PrimeGene, China), IFN-γ (1000 U/ml; PrimeGene, China), prostaglandin E2 (PGE2, 250 ng/ml; PeproTech, USA), R848 (1 µg/ml; InvivoGen, USA), and polyinosinic-polycytidylic acid (20 ng/ml; poly(I:C), InvivoGen, USA) and incubated for 20–24 h.

#### DC vaccine quality testing

The expression of HLA-DR, HLA-ABC, CD11c, CD1c, CD141, CD54, CD70, CD80, CD83, CD86, CD197 (CCR7), and CD274 (PD-L1) on these cells was detected by flow cytometry, and the concentrations of IL-12p70 in culture supernatants were measured by cytometric bead array (CBA,BD Biosciences, USA) to ensure that the cells were predominantly mature DCs. Mature DCs were harvested, washed and counted, and a proportion of the viable cells were stained with trypan blue staining. Following final testing for identity, sterility and endotoxins, 1–3 × 10^7^ DCs were resuspended in 2.5 ml of normal saline containing 1% human albumin (Grifols, S.A.) for clinical use. The first dose was given fresh, and the remaining DC aliquots (5–10 × 10^6^ DCs per dose) were frozen in liquid nitrogen, thawed, and washed 2–3 h before each administration.

### Outcome measures

#### Safety assay

Safety was monitored in all patients during the Neo-DCVac administration period and throughout the treatment. Safety was assessed by an evaluation of the incidence of clinical adverse events, which were graded with the use of the National Cancer Institute Common Terminology Criteria for Adverse Events, version 4.0.

#### Tumor response assay

Tumor response was assessed after each vaccination cycle and included CT of the chest and upper abdomen, magnetic resonance imaging of the head, and bone scintigraphy. The tumor response (CR, PR, SD, or PD) was evaluated per RECIST, version 1.1.^47^ PFS was defined as the duration from the initiation of the therapy to the date of disease progression, intolerable side effects, or death from any cause. OS was defined as the duration from the initiation of therapy to the date of death from any cause.

### Analysis of T-cell responses to neoantigens

#### Patient sample

Patient autologous PBMCs were used to detect the response of T cells against neoantigens in vitro. Heparinized blood samples were obtained from patients after each vaccination cycle, and PBMCs were isolated by Ficoll density-gradient centrifugation.

#### PBMC stimulation

For in vitro stimulation, PBMCs were aliquoted into 24-well cell culture plates at 5 × 10^6^ cells per well with individual neoantigen mutant/WT peptide(10 μg/ml) or mutant/WT peptide-pulsed DCs. The ratio of DC to PBMC was 1:10. The culture medium consisted of AIM-V medium, 10% FBS, and IL-15 (5 ng/ml; PeproTech). On day 3, low-dose IL-2 (10 U/ml; Shandong Quangang Pharmaceutical Co., Ltd., China) was added. Half of the medium was changed and supplemented with fresh medium containing fetal calf serum (FCS), the corresponding peptide and IL-2 at 3-day intervals. After 10–21 days, followed by a 20–24 h restimulation with the peptide or DCs, the specific T-cell responses against each peptide were tested by enzyme-linked immune absorbent spot (ELISpot) and flow cytometry.

#### IFN-γ ELISpot assay

An IFN-γ ELISpot Kit (Dakewei, China) was used to detect IFN-γ release from PBMCs after stimulation with mutant/WT peptides or mutant/WT peptide-pulsed DCs, as previously reported.^19,27^ Briefly, peptide-stimulated PBMCs or peptide-pulsed DC-stimulated PBMCs were added to duplicate wells (10^5^ per well) for 16–20 h. At the same time, no peptide-stimulated PBMCs and 1% phytohemagglutinin (PHA)-stimulated PBMCs were used as negative controls and positive controls, respectively. After washing the plates, the biotinylated antibody was added and incubated at 37 °C for 1 h. After washing the plate again, streptavidin-horseradish peroxidase (HRP) was added and incubated for another 1 h at 37 °C. Then, after washing the plate again, AEC solution mix was added to the plate, and the plate was incubated at room temperature in the dark for ~15–30 min. Finally, deionized water or tap water was added to stop the color rendering. The ELISpot plates were scanned and analyzed using a CTL ImmunoSpot S5 Versa Analyzer (Cellular Technology Ltd., OH, USA). The spots of mutant peptide-stimulated PBMCs or mutant peptide-pulsed DC-stimulated PBMCs greater than twice the negative control or WT-stimulated PBMCs were considered to have positive PBMC reactivity.

#### Flow cytometry for activation markers

The T-cell activation markers 4-1BB (CD137) and OX40 (CD134) were assessed by flow cytometry. Briefly, peptide-stimulated PBMCs or peptide-pulsed DC-stimulated PBMCs were harvested and resuspended in stain buffer (BD Pharmingen). The cells were stained with CD3, CD4, CD8, CD134, and CD137 antibodies (all from BD Bioscience) for 30–40 min at 4 °C in the dark. Then, the cells were washed with cold PBS and resuspended in PBS. All data acquisition was performed using a BD FACSAria II or FACSAria SORP flow cytometer (BD Biosciences), and data analysis was performed using NovoExpress software (ACEA Biosciences).

### Immune correlate analysis

#### Patient samples

Before and after vaccination, patients’ autologous PBMCs were collected and frozen in liquid nitrogen with dimethyl sulfoxide (DMSO) and FBS (Gibco, USA). Patients’ samples were de-identified and assigned a study-specific tracking number.

#### Intracellular cytokine staining

PBMCs were thawed and rested overnight in AIM-V medium supplemented with 10% FBS and 5 ng/ml IL-15. On the next day, PBMCs were stimulated with 10 μg/ml mutant peptides and corresponding WT peptides or 1% PHA (positive control) overnight. Stimulated PBMCs were treated with GolgiStop (BD Biosciences) according to the manufacturer’s recommendations for 4–6 h the following day. After treatment, PBMCs were resuspended in stain buffer and stained with fixable viability stain (FVS), CD3, and CD4 antibodies at 4 °C in the dark. Following 30 min of surface staining, PBMCs were fixed and permeabilized (Fixation/Permeabilization Solution Kit, BD Biosciences). Then, these cells were stained with IFN-γ, IL-2, CD107a, and TNF-α antibodies for 1 h at 4 °C. After washing with permeabilization buffer and fixation (BD Biosciences), cells were analyzed using a BD FACSAria II flow cytometer.

#### CBA analysis of cytokines

The concentrations of cytokines in culture supernatants were measured by CBA according to the manufacturer’s protocol with appropriate diluents. Human IFN-γ Flex Set (Bead E7), human Il-2 Flex Set (Bead A4), and human TNF-α Flex Set (Bead D9) (all from BD Bioscience, USA) were used for the detection of the cytokines IFN-γ, IL-2, and TNF-α, respectively. The samples were run, and fluorescence-activated cell sorting (FACS) data were collected using a FACS Symphony™ A5 (BD Bioscience) flow cytometer and analyzed using FCAP v3.0 array software (Soft Flow, Hungary).

#### TCR sequencing and analysis

Post-treated peripheral blood from patient 17 was collected and incubated with peptides that were ELISpot-positive (*N* = 7) for this patient. Cells were harvested for RNA extraction after 2 weeks of incubation. High-throughput sequencing was performed with the TCR library, which was constructed with cDNA that was reverse transcribed from each RNA sample. CDR3 in the TCR-β chain was thus analyzed with the well-established methods for clone diversity, mean frequency of clones, TCR convergence, and enriched clones.

### Flow cytometry antibodies and procedures

The following human-protein specific flow cytometry antibodies were purchased from BD Biosciences: CD11c-FITC (clone: B-LY6), CD1c-BV-786 (clone: F10/21A3), CD80-BV750 (clone: L307.4), CD83-BV605 (clone: HB15e), CD86-BV711 (clone: 2331), CD274-PE-Cy7 (clone: MIH1), CD197-Percp-Cy5.5 (clone:150503), CD3-APC (clone: UCHT1), CD4-PE-Cy7 (clone: SK3), CD8-APC-Cy7 (clone: Sk1), CD45RO-PerCP-Cy5.5 (clone:UCHL1), CD279-PE (clone:MIH4), CD134-BV421 (clone:ACT35), CD137-BV605 (clone:4B4-1), CD107a-BB700 (clone:H4A3), IL-2-BV605 (clone:MQ1-17H12), IFN-γ-FITC (clone:4 S.B3), and TNF-BV421 (clone:MAb11). BD FACS Symphony™ A5, FACSAria II, and FACSAria SORP flow cytometers were used to perform fluorescent expression analyses, and Novo Express software (ACEA Biosciences) was used for data analyses.

### Statistical analysis

Data from the patients who received at least one dose of Neo-DCVac were included in the safety and clinical effects analyses. Patient characteristics, clinical outcomes and vaccine safety were presented using simple descriptive statistics. Standard RECISTv1.1 guidelines were applied for the analysis of all clinical responses. The DCR was defined as the proportion of CR, PR, and SD for the best clinical response. The product-limit (i.e., Kaplan–Meier) estimator was used to estimate median PFS and OS with corresponding 95% CIs. Differences between averages of variables were compared using a two-tailed *t*-test, and statistical significance was assumed if *p* < 0.05. GraphPad Prism 8 (v8.01) was used to plot survival curves and perform data analyses.

## Supplementary information

supplemental materials

## Data Availability

The original datasets are available from the corresponding author upon request.
