# Supplementary material for: Personalized neoantigen pulsed dendritic cell vaccine for advanced lung cancer
Source: Signal Transduct Target Ther. 2021 Jan 20;6:26. doi: 10.1038/s41392-020-00448-5 (PMC7817684; doi:10.1038/s41392-020-00448-5)
Supplement: Supplementary file 1 — supplemental materials [file 41392_2020_448_MOESM1_ESM.docx]

Supplementary Materials for Personalized neoantigen pulsed dendritic cell vaccine for advanced lung cancer
 Zhenyu Ding ^1,2†^; Qing Li^1,2†^; Rui Zhang ^1†^; Li Xie^1^; Yang Shu^1^; Song Gao^4^; Peipei Wang^1,2^; Xiaoqing Su^1^; Yun Qin^3^; Yuelan Wang^1^; Juemin Fang^4^; Zhongzheng Zhu^4^; Xuyang Xia^1^; Guochao Wei^5^; Hui Wang^4^; Hong Qian^4^; Xianling Guo^4^; Zhibo Gao^5^; Yu Wang^5^; Yuquan Wei^1^; Qing Xu^4^*; Heng Xu ^1^*; Li Yang ^1^*

^1^State Key Laboratory of Biotherapy and Cancer Center, West China Hospital, Sichuan University, and Collaborative Innovation Center for Biotherapy, Chengdu, 610041, China.

^2^Department of Biotherapy, Cancer Center, West China Hospital, Sichuan University, Chengdu, China.

^3^Department of Radiology, West China Hospital, Sichuan University, Chengdu, Sichuan, China.

^4^Department of Oncology, Cancer Center, Shanghai Tenth People’s Hospital, Tongji University, Shanghai, 200072, China

^5^YuceBio Technology Co., Ltd, Shenzhen, China

**Corresponding author:** Qing Xu^4^; Heng Xu ^1^; Li Yang ^1^

E-mail address:

[xuqingmd@tongji.edu.cn](mailto:xuqingmd@tongji.edu.cn);

[xuheng81916@scu.edu.cn](mailto:xuheng81916@scu.edu.cn);

[yl.tracy73@gmail.com](mailto:杨莉yl.tracy73@gmail.com);

**
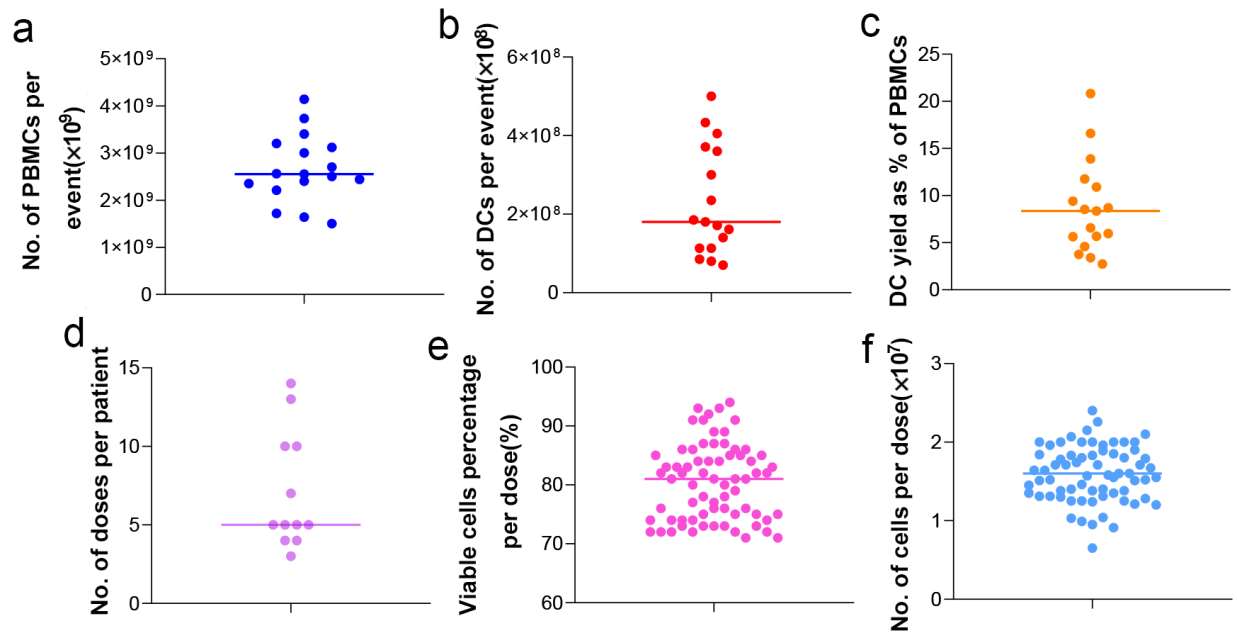
**

**Supplementary Figure 1. Feasibility of Neo-DCVac.** (**a**) The number of peripheral blood mononuclear cells (PBMCs). Each dot represents an events during Neo-DCVac treatment. The lines show the median. (**b**) The number of dendritic cells (DCs). Each dot represents an event during Neo-DCVac treatment. The lines show the median. (**c**) The percentage of DCs yielded from PBMCs. Each dot represents an event during Neo-DCVac treatment. (**d**) Number of Neo-DCVac doses that each patient received. Each dot represents a different patient. (**e**) The percentage of viable cells per dose. Each dot represents a dose. (**f**) The number of viable cells per dose. Each dot represents a dose.


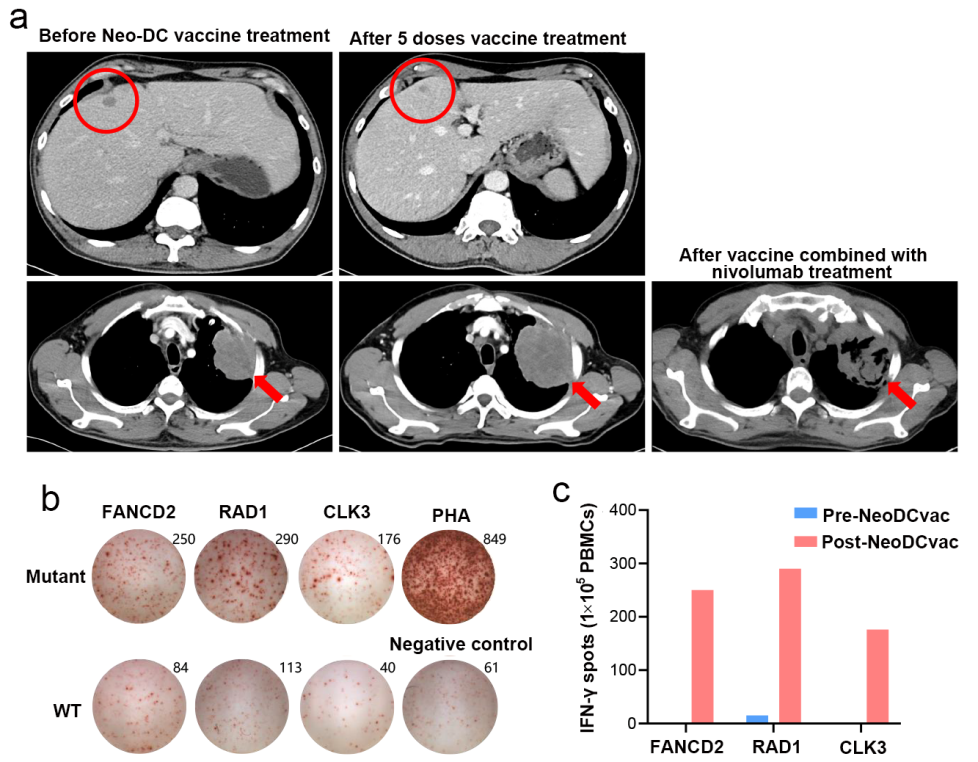


**Supplementary Figure 2. Clinical and immune responses to personalized Neo-DCVac in patient 6 with metastatic lung adenocarcinoma.** (**a**) CT scans were performed before and after personalized immunotherapy, and representative images are shown. Before and after Neo-DCVac, autologous peripheral blood mononuclear cells (PBMCs) were stimulated with 16 candidate mutant peptides for 10 days, after which IFN-γ ELISpot assays were performed to assess the T-cell-specific antigen response. Phytohemagglutinin (PHA) and no peptide stimulation represent positive and negative controls, respectively. (**b**) The IFN-γ ELISpot picture of fold changes of mutant peptides/WT peptides >2 is shown. (**c**) Before and after Neo-DCVac treatment shows that the PBMCs responded to these peptides.

**
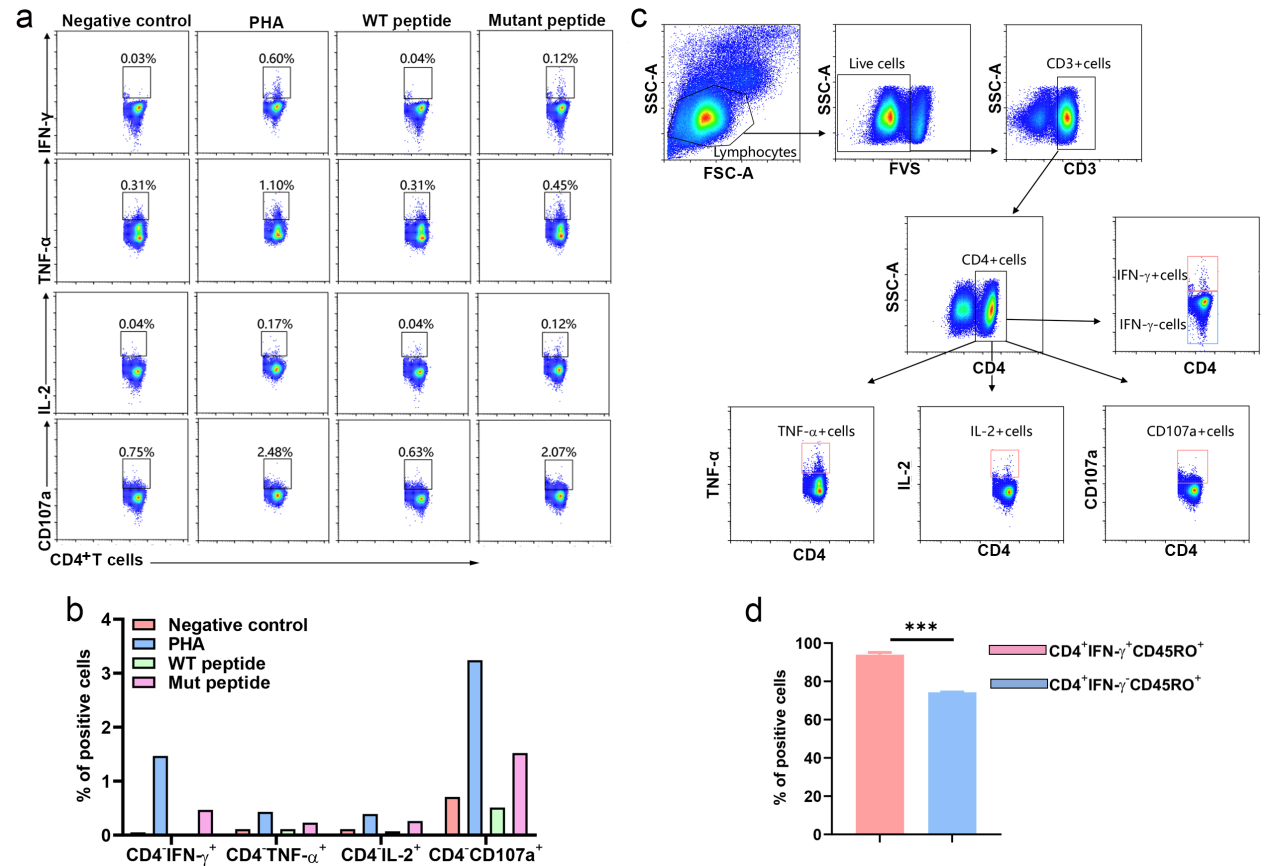
**

**Supplementary Figure 3. Gating strategies used for intracellular cytokine analysis.** Analysis of *ex vivo* T-cell responses to neoantigen peptides after exposure to neoepitopes and corresponding wild-type (WT) peptides overnight using intracellular cytokine staining followed by flow cytometry. Cells were stained using antibodies against FVS, CD3, CD4, CD45RO, PD-1, L-2, IFN-γ, TNF-α and CD107a. ****P*< 0.001


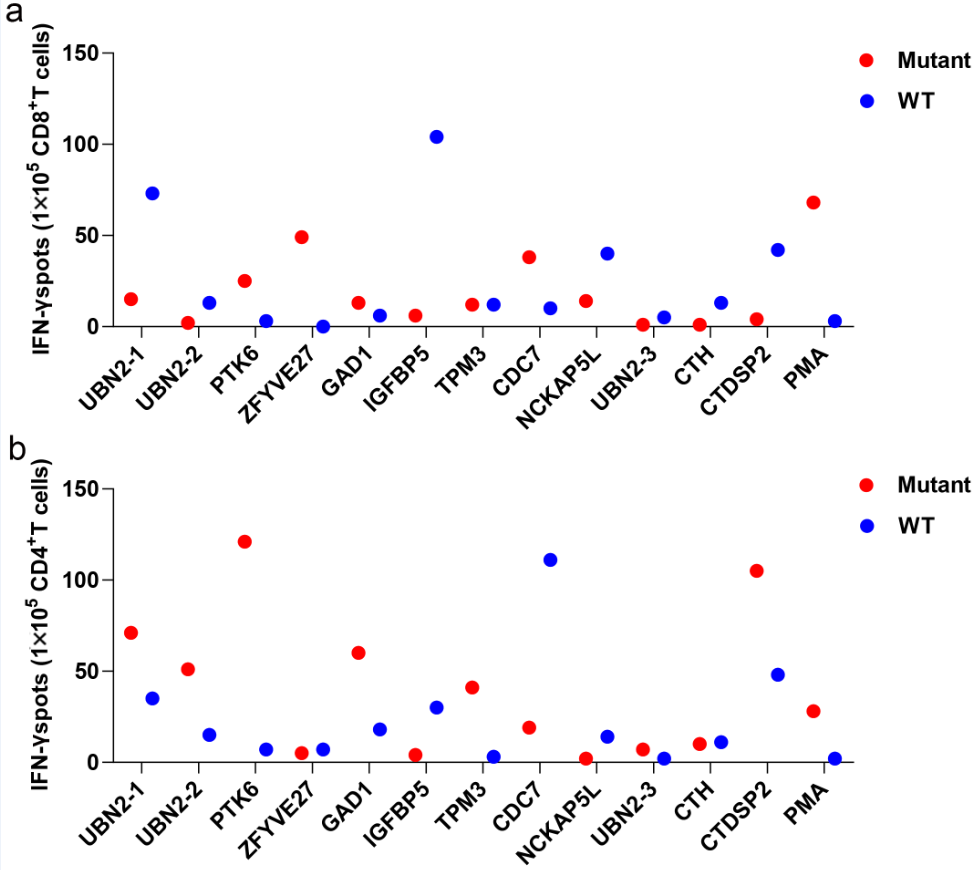


**Supplementary Figure 4. ELISpot assay of patient 15.** Autologous (a) CD8^+^ T cells and (b) CD4^+^ T cells were stimulated with 12 candidate mutant peptides and corresponding wild-type (WT) peptides for 10 days, after which IFN-γ ELISpot assays were performed to assess the T-cell-specific antigen response. One percent phytohemagglutinin (PHA) and no peptide stimulation represent the positive and negative controls, respectively.

**Supplementary Table 1. Neoepitope analysis of clinical study patients. Patient identification number, total number of nonsynonymous mutations, number of neoepitopes and peptides in vaccine.**

| Patient ID | Nonsynonymous mutations | HLA I binders | number of expressed neoepitopes HLA I | HLA II binders | number of expressed neoepitopes HLA II | peptides in vaccine HLA I | peptides in vaccine HLA II | peptides in vaccine |
| --- | --- | --- | --- | --- | --- | --- | --- | --- |
| 1 | 808 | 344 | 151 | 1718 | 617 | 13 | 0 | 13 |
| 2 | 326 | 252 | 116 | 200 | 70 | 15 | 0 | 15 |
| 4 | 344 | 870 | 295 | 1236 | 363 | 25 | 5 | 30 |
| 6 | 262 | 363 | 137 | 21 | 16 | 15 | 1 | 16 |
| 7 | 80 | 102 | 48 | 527 | 247 | 9 | 6 | 15 |
| 8 | 130 | 35 | 10 | 58 | 36 | 15 | 3 | 18 |
| 12 | 459 | 714 | 319 | 1177 | 358 | 16 | 6 | 22 |
| 14 | 312 | 702 | 346 | 7 | 7 | 15 | 2 | 17 |
| 15 | 181 | 180 | 97 | 66 | 26 | 9 | 3 | 12 |
| 16 | 217 | 276 | 122 | 627 | 269 | 10 | 7 | 17 |
| 17 | 315 | 285 | 112 | 694 | 229 | 12 | 3 | 15 |
| 18 | 482 | 747 | 350 | 439 | 191 | 14 | 3 | 17 |

**Supplementary Table 2: Objective responses, disease control and progressive disease**

|  | Objective responses  (n, %) | Disease control  (n, %) | Progressive disease  (n, %) |
| --- | --- | --- | --- |
| All patients (n=12) | 3 (25%) | 9 (75%) | 3 (25%) |
| T-cell response neoantigens |  |  |  |
| >8 (n=6) | 1 (16.7%) | 5 (83.3%) | 1(16.7%) |
| 3-8 (n=4) | 1(20%) | 2(50%) | 2(50%) |
| Not available (n=2) | 1(50%) | 2 (100%) | 0 |
| Combination of Neo-DCVac and ICIs (n=5) | 2 (40%) | 5 (100%) | 0 |
| Not combination of Neo-DCVac and ICIs (n=7) | 1(14.3%) | 4(57.1%) | 3(42.9%) |

**Supplemental Table 3. Candidate HLA-binding peptides for patient 1**

| HLA | Mutant peptide | Wild type peptide | Gene | Neoepitope Score ^a^ | Wide type Score ^b^ |
| --- | --- | --- | --- | --- | --- |
| HLA-B*40:01 | EENENSANL | EENENSANQ | MARCH6 | 177.4 | 27203.8 |
| HLA-B*40:01 | AETYARYRYL | AETYARYRYP | B4GALNT1 | 149.8 | 15355.1 |
| HLA-C*07:02 | LRMAVSHMM | LRMAVSHMK | ARNT | 58.5 | 4942.7 |
| HLA-C*07:02 | FYNSDYVAAL | FYNSDYVAAR | CHPF | 159.8 | 4166.7 |
| HLA-B*40:01 | YEFKECGKAF | YECKECGKAF | ZNF44 | 184.6 | 1445.8 |
| HLA-B*40:01 | KEWMRLCTSL | KEWMRLCTSV | PMPCB | 80.5 | 476.4 |
| HLA-C*07:02 | MRSSAATEL | MHSSAATEL | QSER1 | 65.5 | 381.3 |
| HLA-C*07:02 | HRRRRWVLL | HRRRRWVRL | DYSF | 66.9 | 211.6 |
| HLA-A*24:50 | KMPKFSMPVF | KMPKFSMPGF | AHNAK | 132.1 | 250.9 |
| HLA-B*40:01 | REPFVLMQL | REPFVRMQL | CUX1 | 58.7 | 110.1 |
| HLA-B*40:01 | VEFYSLNPSL | VELYSLNPSL | WDR3 | 38.2 | 60.3 |
| HLA-B*40:01 | AEIFYQIVDL | AEILDQIVDL | SPEF2 | 44.9 | 70.7 |
| HLA-B*40:01 | LEYFLSFVVL | LEYILSFVVL | GPAM | 27.5 | 40.9 |

**^a^** MHC affinity score of MUT peptide; **^b^** MHC affinity score of WT peptide. Immunogenic peptides are marked in red.

**Supplemental Table 4. Candidate HLA-binding peptides for patient 15**

| HLA | Mutant peptide | Wild type peptide | Gene | Neoepitope Score ^a^ | Wide type Score ^b^ |
| --- | --- | --- | --- | --- | --- |
| HLA-B*27:05 | LRLHSFPTML | LPLHSFPTML | UBN2-2 | 42 | 17166.9 |
| HLA-A*02:07 | FLGNVEFFL | FLGNVEFFR | ZFYVE27 | 52.5 | 14130.4 |
| HLA-A*03:01 | VPSIHRPLEK | VPSIHRPLEQ | NCKAP5L | 205.8 | 17940.2 |
| HLA-A*03:01 | LRLHSFPTMLK | LPLHSFPTMLK | UBN2 | 17.8 | 1449.4 |
| HLA-B*27:05 | ERWAQGLRCL | ERCAQGLRCL | IGFBP5 | 84.6 | 2497.7 |
| DRB1_0401 | NDRYVLGISDTLIRL | NDRDVLGISDTLIRL | CTH | 111.94 | 1004.55 |
| HLA-B*27:05 | KRIHQFGIVHL | KRIHQFGIVHR | CDC7 | 151.1 | 808.5 |
| DRB1_0401 | NIFKALFCSFRAQHV | NIFKALFCCFRAQHV | CTDSP2 | 105.03 | 307.64 |
| HLA-A*03:01 | RAFKERQSSK | SAFKERQSSK | GAD1 | 73.7 | 214.1 |
| HLA-A*03:01 | RDTQAVRLYK | RDTQAVRHYK | PTK6 | 24.7 | 66.7 |
| HLA-C*02:02 | HAINDMTSI | HALNDMTSI | TPM3 | 116.3 | 303.8 |
| DRB1_0401 | MVKTLRLHSFPTMLKECSP | MVKTLPLHSFPTMLKECSP | UBN2-1 | 89.69 | 136.28 |

**^a^** MHC affinity score of MUT peptide; **^b^** MHC affinity score of WT peptide. Immunogenic peptides are marked in red.

**Supplemental Table 5. Candidate HLA-binding peptides for patient 17**

| HLA | Mutant peptide | Wild type peptide | Gene | Neoepitope Score ^a^ | Wide type Score ^b^ |
| --- | --- | --- | --- | --- | --- |
| HLA-A*11:01 | STVRNIASR | SRVRNIASR | PLCH1 | 48 | 22724.5 |
| HLA-B*40:01 | HELQLKIGIL | HGLQLKIGIL | PHF20L1 | 69.1 | 29758.3 |
| HLA-A*11:01 | LSLVFVDKPK | LSLVFVDKPE | ACSL1 | 64.6 | 19486.4 |
| HLA-A*11:01 | SVSPAFSK | SGSPAFSK | 2-Mar | 95.7 | 3637.1 |
| HLA-B*39:01 | WPAFASGLL | GPAFASGLL | DENND1A | 166 | 2983 |
| HLA-C*07:02 | LRPQLYGVF | LGPQLYGVF | CHKB | 190.4 | 2941.1 |
| HLA-C*07:02 | SRAKRRRTL | SRAKRQKDF | DNAJC1 | 153.3 | 1734 |
| HLA-B*40:01 | GETPALDEV | GEPPALDEV | CPD | 74.2 | 592.2 |
| HLA-A*11:01 | MVFAAKAMK | MGFAAKAMK | CHMP4C | 17.6 | 109.6 |
| DRB1_0803 | RNMVFAAKAMKS | RNMGFAAKAMKS | CHMP4C | 32.85 | 203.01 |
| DRB1_0803 | LSLVFVDKPKKAKLL | LSLVFVDKPEKAKLL | ACSL1 | 45.94 | 262.77 |
| DRB1_0803 | PDIFLGLRKIEAE | PDIFPGLRKIEAE | SGPL1 | 96.21 | 520.41 |
| HLA-A*11:01 | SSSRSLPNK | SSRRSLPNK | KIF13A | 16.1 | 65.1 |
| HLA-A*11:01 | TVFSMQSNR | TVLSMQSNR | PPP1R7 | 74 | 223.9 |
| HLA-A*11:01 | VVGACGVGK | VVGAGGVGK | KRAS | 90.6 | 139.3 |

**^a^** MHC affinity score of MUT peptide; **^b^** MHC affinity score of WT peptide. Immunogenic peptides are marked in red.

**Supplemental Table 6. Candidate HLA-binding peptides for patient 6**

| HLA | Mutant peptide | Wild type peptide | Gene | Neoepitope Score ^a^ | Wide type Score ^b^ |
| --- | --- | --- | --- | --- | --- |
| HLA-A*11:01 | MSLPLTARWK | MSLPLTARWE | CPOX | 18.4 | 10949.6 |
| HLA-B*15:01 | TQIITGSDEF | TQIITGSDES | CLSPN | 27.7 | 9504.5 |
| HLA-A*11:01 | ISLPGVSSTK | ISLPGVSSTE | EFCAB14 | 62 | 18959.4 |
| HLA-B*15:01 | INKYGVSLIY | INKYGVSLID | MYO7A | 291.8 | 32815.3 |
| HLA-A*11:01 | SQFETLYNEHK | SEFETLYNEHK | CLK3 | 232.5 | 11829.2 |
| HLA-B*15:01 | RSRLGPPDVY | RPRLGPPDVY | MED12L | 246.3 | 5641.5 |
| HLA-B*15:01 | MSILSGCDY | MCILSGCDY | EXO1 | 78.9 | 1176.6 |
| HLA-A*11:01 | TVLSPTKKK | TVLSPTKKR | CD3EAP | 62.6 | 578.4 |
| HLA-A*11:01 | YGVSLIYPK | YGVSLIDPK | MYO7A | 71.8 | 398.3 |
| HLA-DRB1*14:54 | CHALRFLHKNQLTHT | CHALRFLHENQLTHT | CLK3 | 72.86 | 334.49 |
| HLA-B*15:01 | MKSFQKTPHY | IKSFQKTPHY | SLC15A2 | 124 | 377.5 |
| HLA-B*15:01 | VVMKFPNGF | VVMKFPDGF | ABCF3 | 179.7 | 543 |
| HLA-A*11:01 | CSVSPSVSR | CSVSPSGSR | FAAP100 | 145.9 | 368.6 |
| HLA-A*11:01 | QVNRYNISLLK | QVNRYKISLLK | RAD1 | 50.1 | 121 |
| HLA-B*15:01 | TLNNCTEAF | TLNNCREAF | FANCD2 | 45.8 | 105.3 |
| HLA-A*11:01 | SMRLPLVLDK | SMRLPLVSDK | GREB1L | 42.3 | 89.8 |

**^a^** MHC affinity score of MUT peptide; **^b^** MHC affinity score of WT peptide. Immunogenic peptides are marked in red.

**Supplemental Table 7. Candidate HLA-binding peptides for patient 2**

| HLA | Mutant peptide | Wild type peptide | Gene | Neoepitope Score ^a^ | Wide type Score ^b^ |
| --- | --- | --- | --- | --- | --- |
| HLA-B*40:01 | AEAMEILLSL | AEAMEILLSR | SPATS2 | 13.3 | 14198 |
| HLA-B*40:01 | AEFEVPKLVL | AEFEVPKLVQ | FBXW8 | 22.2 | 9603.2 |
| HLA-C*15:02 | STLPPSRSL | STLPPSRSR | NPHP4 | 80.4 | 16032.8 |
| HLA-C*08:01 | LADSSGNLL | LEDSSGNLL | TP53 | 47.9 | 4494.8 |
| HLA-A*24:02 | AWQLPQYALF | AWQLPQYALV | SLC15A2 | 30.6 | 1621.2 |
| HLA-C*08:01 | RATGGTAVM | RGTGGTAVM | IDO2 | 169.1 | 1782.3 |
| HLA-A*24:02 | RQPPVSLPF | REPPVSLPF | PIGO | 145 | 1525.8 |
| HLA-B*40:01 | AAEHQTTSPL | AAEHQTTSPV | ZNF551 | 89.3 | 677.7 |
| HLA-B*40:01 | ERELELRDTL | ERELELRDRL | CCDC69 | 147.7 | 912.4 |
| HLA-C*15:02 | FSSLNSGQI | VSSLNSGQI | MAN2A1 | 114.4 | 581.9 |
| HLA-B*40:01 | AEHQTTSPL | AEHQTTSPV | ZNF551 | 13.9 | 67.9 |
| HLA-B*40:01 | LEQILPPRL | LERILPPRL | TBC1D32 | 145.5 | 707.6 |
| DRB1_1501 | LFRQFTFVNSKTVT | LFRQVTFVNSKTVT | ZNF300 | 76.46 | 195.14 |
| HLA-B*40:01 | SEHFSRFGTL | NEHFSRFGTL | RBM26 | 45.9 | 113.4 |
| DRB1_1501 | NVQMLFSNPLYEPV | NVQMLFSNPLDEPV | TGM3 | 1023.48 | 1464.59 |

**^a^** MHC affinity score of MUT peptide; **^b^** MHC affinity score of WT peptide. Immunogenic peptides are marked in red.

**Supplemental Table 8. Candidate HLA-binding peptides for patient 4**

| HLA | Mutant peptide | Wild type peptide | Gene | Neoepitope Score ^a^ | Wide type Score ^b^ |
| --- | --- | --- | --- | --- | --- |
| HLA-A*02:03 | MLCSDSSLQL | MLCSDSSLQR | CARD6 | 33.1 | 19965.5 |
| HLA-A*02:03 | HLFPTMPRHI | HLFPTMPRHN | FADS2 | 28.7 | 14961.4 |
| HLA-B*39:01 | WKENTPWTL | WKENTPWTS | GPATCH2L | 30.1 | 12720.1 |
| HLA-B*39:01 | CRVGYFRAL | CRVGYFRAR | EPHB4 | 130.8 | 14071.9 |
| HLA-B*35:01 | FPRAKMYFM | FPRAKMYFV | TENM2 | 40.7 | 2230.2 |
| HLA-A*02:03 | ALTSDMIPRL | AFTSDMIPRL | ANO6 | 42.1 | 1753 |
| HLA-B*39:01 | SQLALDRPL | SQLALDRPF | TNKS1BP1 | 83.3 | 1746.4 |
| HLA-C*15:02 | VSFGGNLLM | VSCGGNLLM | FUCA2 | 59.3 | 738.7 |
| HLA-A*02:01 | FLSGSVFL | ILSGSVFL | SNX14 | 32.2 | 337.8 |
| HLA-A*02:03 | KVYDYVRKIV | KVYDYVRKIL | THAP9 | 86.1 | 722.8 |
| HLA-A*02:03 | MVAAAGMLL | MAAAAGMLL | CYP51A1 | 62.7 | 525 |
| HLA-B*35:01 | MPTLEHTTTSF | MPTLEHTTKSF | FAM83B | 52.6 | 396.1 |
| HLA-A*02:03 | ILIASRDIV | ILIASRDIA | KMT5A | 82.6 | 553.2 |
| HLA-A*02:03 | FMQNNPSIPA | FIQNNPSIPA | RREB1 | 21 | 136.6 |
| HLA-A*02:03 | RVPFRLLVNV | RVPFRLLVNA | LRRC24 | 55.3 | 341.9 |
| HLA-A*02:01 | TGLSHELWRV | TGLSHELCRV | NCKIPSD | 122.6 | 662.8 |
| HLA-B*35:01 | LPMTRGAGM | LPTTRGAGM | RPTOR | 28.1 | 148.4 |
| DRB1_1101 | FLQMLLGHRG | FSQMLLGHRG | KIAA0895L | 29.96 | 151.43 |
| DRB1_1101 | FLSAKHRRQVYLQI | FLSAKHQRQVYLQI | ASPM | 33.96 | 134.6 |
| DRB1_1101 | VGYFRALTDPRGAP | VGYFRARTDPRGAP | EPHB4 | 30.47 | 118.86 |
| DRB1_1101 | HDFFESLHRRQAFHI | HDFFESLHRGQAFHI | VPS13D | 28.86 | 102.51 |
| HLA-A*02:03 | FSAYWLQLV | FSAYWHQLV | MKRN3 | 16.6 | 52.3 |
| HLA-A*02:03 | YLHFLELHT | YLHFLEPHT | KIF1A | 92.6 | 265.6 |
| HLA-A*02:01 | QLMSECLLYYI | PLMSECLLYYI | KIF1A | 30.9 | 75.1 |
| DRB1_1101 | KDFNLLRLLGANAF | KDFNLLRWLGANAF | GUSB | 47.78 | 100.25 |
| HLA-A*02:03 | VLRHHLFAA | VLRHQLFAA | ATN1 | 11.3 | 21.9 |
| HLA-A*02:03 | CLQLSTLPGA | CLQLSTVPGA | C2orf69 | 81.3 | 157.3 |
| HLA-A*02:03 | LLSGKFQGPI | LLSGKFQGLI | CDC25C | 28.8 | 50.8 |
| HLA-A*02:01 | FTLHMLLDL | FTLQMLLDL | TP53 | 126.1 | 210.4 |
| HLA-A*02:03 | FVSSIFPV | FVSCIFPV | COL16A1 | 27.4 | 44.4 |

**^a^** MHC affinity score of MUT peptide; **^b^** MHC affinity score of WT peptide. Immunogenic peptides are marked in red.

**Supplemental Table 9. Candidate HLA-binding peptides for patient 7**

| HLA | Mutant peptide | Wild type peptide | Gene | Neoepitope Score ^a^ | Wide type Score ^b^ |
| --- | --- | --- | --- | --- | --- |
| HLA-B*15:02 | LAAFGHMLY | LAAFGHMLC | INPP5J | 37.1 | 19701.8 |
| HLA-A*11:01 | RLAAFGHMLY | RLAAFGHMLC | INPP5J | 68.9 | 17591.9 |
| HLA-B*15:02 | CAKAFPKAY | CGKAFPKAY | E4F1 | 219.6 | 2734.3 |
| DRB1_1501 | FGHMLYFLNCHLP | FGHMLCFLNCHLP | INPP5J | 44.31 | 226.29 |
| DRB1_1202 | KITDFGRAKLL | KITDFGLAKLL | EGFR | 45.38 | 216.69 |
| HLA-A*11:01 | KTFNWSSALNK | KAFNWSSALNK | ZNF506 | 26.2 | 97.4 |
| HLA-A*11:01 | GMHMGQLLK | GMRMGQLLK | DDX60L | 51.8 | 189.1 |
| HLA-A*11:01 | KRSCKGALYK | ERSCKGALYK | BBX | 78.1 | 253.2 |
| DRB1_1202 | TNILPVNQPVRLGAS | TNILPVNQPVRPGAS | ADNP2 | 96.97 | 309.99 |
| HLA-A*11:01 | KSAKSDAPK | KSTKSDAPK | SRPRA | 70.8 | 180.2 |
| DRB1_1202 | NILELASGAVLR | NILEVASGAVLR | TBL3 | 78.08 | 190.81 |
| DRB1_1202 | HHKFHSQRHRSKK | HHKFHSQRHGSKK | TMEM120A | 42.85 | 104.62 |
| DRB1_1202 | ACAQCAKAFPKAYL | ACAQCGKAFPKAYL | E4F1 | 112 | 232.79 |
| HLA-B*15:02 | HSIHPYTVF | HSIRPYTVF | QRICH2 | 36.3 | 61.7 |
| HLA-A*11:01 | CAQCAKAFPK | CAQCGKAFPK | E4F1 | 85.7 | 111.5 |

**^a^** MHC affinity score of MUT peptide; **^b^** MHC affinity score of WT peptide.

**Supplemental Table 10. Candidate HLA-binding peptides for patient 8**

| HLA | Mutant peptide | Wild type peptide | Gene | Neoepitope Score ^a^ | Wide type Score ^b^ |
| --- | --- | --- | --- | --- | --- |
| HLA-A*11:03 | TIDPASEFMFK | TIDPASEFMFE | FOXF1 | 27.2 | 16418.8 |
| HLA-A*11:03 | IPVAIKTSPK | IPVAIKELRE | EGFR | 158.2 | 31132.7 |
| HLA-A*11:03 | MVKTRASLK | MVKTRAKVL | SACS | 210.7 | 35072.1 |
| HLA-A*11:03 | AGAGPLPSSK | AGAGPLPSSE | WRAP73 | 204.8 | 28934.3 |
| HLA-A*11:03 | AIKTSPKANK | AIKELREATS | EGFR | 348.6 | 36310.4 |
| HLA-B*15:01 | LANKPIAPNF | PANKPIAPNF | ANKRD17 | 81.2 | 7452.9 |
| HLA-B*15:01 | LANKPIAPNF | PANKPIAPNF | ANKRD17 | 81.2 | 7452.9 |
| HLA-A*11:03 | KIPVAIKTSPK | KIPVAIKELRE | EGFR | 295.3 | 26745.3 |
| HLA-A*24:02 | GYPYPFSGIF | GHPYPFSGIF | DESI2 | 60.5 | 3933.7 |
| HLA-B*15:01 | AIKRALQQF | AIERALQQF | SUPT6H | 94 | 2466 |
| HLA-C*07:02 | FRRFPCLSL | FKRFPCLSL | RHOA | 51.9 | 832.6 |
| HLA-DRB1*04:05 | VRDFSQVHPLAVLL | VRDSSQVHPLAVLL | DHX30 | 177.02 | 2181.12 |
| HLA-B*15:01 | RLQCSGMIM | RLQCSGMII | SRP9 | 54.7 | 574.7 |
| HLA-DRB1*04:05 | AQIFVRVLDTNDNS | AQISVRVLDTNDNS | PCDHAC2 | 109.28 | 818.32 |
| HLA-B*15:01 | LIMQLMPF | LITQLMPF | EGFR | 217.5 | 1325.9 |
| HLA-B*15:01 | MQLMPFGCLL | TQLMPFGCLL | EGFR | 64.3 | 301.6 |
| HLA-B*15:01 | MQLMPFGCL | TQLMPFGCL | EGFR | 143.3 | 642.2 |
| HLA-DRB1*04:05 | GWKVLLEQVSHLLGW | GWKVPLEQVSHLLGW | SLC22A31 | 308.29 | 1325.95 |
| HLA-A*11:03 | CQNSVHLANK | CQNSVHPANK | ANKRD17 | 156.3 | 444.6 |

**^a^** MHC affinity score of MUT peptide; **^b^** MHC affinity score of WT peptide. Immunogenic peptides are marked in red.

**Supplemental Table 11. Candidate HLA-binding peptides for patient 12**

| HLA | Mutant peptide | Wild type peptide | Gene | Neoepitope Score a | Wide type Score b |
| --- | --- | --- | --- | --- | --- |
| HLA-C*03:04 | KAYTGTILM | EGVYRNNID | PTEN | 11.5 | 47631.6 |
| HLA-B*40:01 | FEHSLSPTL | FAHSLSPTL | BIRC2 | 9 | 7077.4 |
| HLA-A*02:01 | SLDEAALPEL | SRDEAALPEL | RETREG3 | 40.2 | 25417.3 |
| HLA-C*03:04 | FANSAIPGSL | FANSAIPGSP | RNF115 | 34.5 | 20024.8 |
| HLA-B*40:01 | GETCAVASNM | GGTCAVASNM | NOTCH2 | 67.9 | 33553.1 |
| HLA-A*02:01 | FLYWHLEDLNV | FLYWHLEDLNG | MFSD6 | 26.9 | 2934.2 |
| HLA-A*02:01 | KLMLKLTAL | KLKLKLTAL | LLGL2 | 19.8 | 1456.9 |
| HLA-A*02:01 | ALQPQAPTSV | ASQPQAPTSV | POLE4 | 317.1 | 16138.3 |
| HLA-C*03:04 | KLRSVSVYL | KLRSVSVDL | SNX5 | 17.2 | 685.3 |
| HLA-A*24:02 | KWGLALAAVF | KWGLALAAVV | SCAP | 32.5 | 1156.1 |
| HLA-A*02:01 | QEVGAAVGEL | QEVGAAGGEL | STAT6 | 59.8 | 847.3 |
| HLA-B*40:01 | SEPMFHSESL | SEPKFHSESL | ACRBP | 32.3 | 329.2 |
| DRB1_1001 | IHITLLLSTQLYY | IHIIATLLLSTQL | SIDT2 | 40.71 | 290.11 |
| HLA-A*02:01 | LVLMCGPPPI | LVLMCGPPPM | CYB5R3 | 19.7 | 106 |
| HLA-A*02:01 | FTVLSSLLMSV | VTVLSSLLMSV | SCAP | 22.9 | 122.8 |
| HLA-A*02:01 | FSIIHITLL | FSIIHIIAT | SIDT2 | 22.9 | 122.8 |
| DRB1_0803 | MRLILNLRR | MRLILNLCR | FAM120AOS | 30 | 146.47 |
| DRB1_0803 | RMSFVKGCMKYIF | RMSFVKGWGAEYR | SMAD2 | 43.16 | 203.83 |
| DRB1_0803 | FRVRALRPRDL | FRVRALRPGDL | TRIM3 | 37.36 | 149.86 |
| DRB1_0803 | RPQLLKVKRKKPR | RPQLSKVKRKKPR | NHEJ1 | 48.22 | 147.79 |
| DRB1_1001 | FKQLRALCVSSC | FKQVRALCVSSC | ORAOV1 | 40.26 | 120.66 |
| HLA-B*40:01 | FQNGESATL | FQNGESATH | ZMYM2 | 84.6 | 203.2 |

**^a^** MHC affinity score of MUT peptide; **^b^** MHC affinity score of WT peptide. Immunogenic peptides are marked in red.

**Supplemental Table 12. Candidate HLA-binding peptides for patient 14**

| HLA | Mutant peptide | Wild type peptide | Gene | Neoepitope Score ^a^ | Wide type Score ^b^ |
| --- | --- | --- | --- | --- | --- |
| HLA-B*58:01 | VSSPKVLQW | VSSPKVLQR | CACNB4 | 4.8 | 17064.3 |
| HLA-B*58:01 | KVAGRLLLW | KVAGRLLLR | CCDC180 | 8.1 | 20824.5 |
| HLA-A*33:03 | HVLDWLKYR | HVLDWLKYG | WSCD1 | 7 | 14430.4 |
| HLA-A*33:03 | TVKEWYVKER | TVKEWYVKEG | DBT | 40.3 | 33556 |
| HLA-B*58:01 | LPSTSLQFIW | LPSTSLQFIG | ATXN1L | 3.2 | 636.9 |
| HLA-B*58:01 | LPSTSLQFIW | LPSTSLQFIG | ATXN1L | 3.2 | 636.9 |
| HLA-A*24:02 | EYYPMTEQF | EHYPMTEQF | SCN3A | 25.8 | 4211.2 |
| HLA-A*33:03 | CVQRACHPR | CDQRACHPR | TENM4 | 24.1 | 2593.1 |
| HLA-A*33:03 | NTSTLQCIR | NRSTLQCIR | WDR3 | 47.3 | 4934.1 |
| HLA-B*58:01 | VSWRILKVLLW | VGWRILKVLLW | TMEM117 | 48.9 | 751.9 |
| HLA-C*03:02 | VAIAATLFV | VGIAATLFV | GRM4 | 79.3 | 886.7 |
| HLA-A*33:03 | LVRNSFEVR | LGRNSFEVR | TP53 | 76.5 | 745.5 |
| HLA-A*33:03 | QYGVFYSCAR | QNGVFYSCAR | LRRIQ1 | 76.9 | 556 |
| HLA-DQA10501-DQB10602 | GWMGAACVQRA | GWMGAACDQRA | TENM4 | 44.8 | 262.54 |
| HLA-B*58:01 | LSSNILTDWTF | LSSNILTDWTI | GLB1 | 33.8 | 189.8 |
| HLA-B*15:01 | MLVDLQYSM | MLVDLQYSL | DOCK10 | 57.2 | 319.6 |
| HLA-B*15:01 | KPQMDLFSCM | KPQLDLFSCM | BRINP1 | 36.8 | 193.2 |
| DRB1_1501 | LIFAYKHALVN | LTFAYKHALVN | TPP2 | 73.19 | 288.7 |

**^a^** MHC affinity score of MUT peptide; **^b^** MHC affinity score of WT peptide. Immunogenic peptides are marked in red.

**Supplemental Table 13. Candidate HLA-binding peptides for patient 16**

| HLA | Mutant peptide | Wild type peptide | Gene | Neoepitope Score ^a^ | Wide type Score ^b^ |
| --- | --- | --- | --- | --- | --- |
| HLA-A*11:01 | ATSPASASK | ATSPASASF | NR4A1 | 11.9 | 5155.7 |
| HLA-A*11:01 | CSTSISNFK | CSTSISNFE | GUCD1 | 24.1 | 10390.9 |
| HLA-B*40:01 | SEHGFGPSL | SEHGFGPIT | MSRA | 8.9 | 1200.5 |
| HLA-C*08:01 | AAASATLAL | AAASATLAS | SOX30 | 68.5 | 8813.8 |
| HLA-C*07:02 | SYFRGSYSY | PYFRGSYSY | SMOX | 75 | 2131.2 |
| DRB1_1201 | ARPVEWLGRCILDA | ARPDEWLGRCILDA | CHPF | 74.77 | 1671.76 |
| DRB1_1201 | EFTDLLSFIGRIR | EFTDPLSFIGRIR | KDM5A | 10.42 | 162.29 |
| DRB1_1201 | LCKMINLSKPDTI | LCKMINLSEPDTI | PLS1 | 24.23 | 260.03 |
| DRB1_1201 | LCTDVALPLIVHNIQ | LCTDVAPPLIVHNIQ | JAK1 | 84.51 | 772.41 |
| HLA-B*40:01 | VEWLGRCIL | DEWLGRCIL | CHPF | 21.5 | 169.1 |
| DRB1_1201 | VGLAVNSAVLYVLL | VGLAGNSAVLYVLL | NPBWR1 | 49.15 | 285.39 |
| HLA-DQA10508-DQB10301 | HGFGPSLPTSGRDRL | HGFGPITTDIREGQT | MSRA | 84.99 | 451.3 |
| DRB1_1201 | CWISFDTHLLY | CWISSDTHLLY | CALCRL | 39.17 | 172.87 |
| HLA-B*40:01 | SEIISFKSL | SEIISFKSM | FEZ1 | 24.9 | 106.4 |
| HLA-A*02:10 | YSLPNAPTV | YSLPNAPTL | TPGS2 | 52.8 | 208.3 |
| HLA-A*11:01 | ISFDTHLLY | ISSDTHLLY | CALCRL | 61.8 | 209.2 |
| HLA-A*02:10 | MLICCCCTL | VLICCCCTL | SLC12A4 | 92.4 | 244.6 |

**^a^** MHC affinity score of MUT peptide; **^b^** MHC affinity score of WT peptide. Immunogenic peptides are marked in red.

**Supplemental Table 14. Candidate HLA-binding peptides for patient 18**

| HLA | Mutant peptide | Wild type peptide | Gene | Neoepitope Score ^a^ | Wide type Score ^b^ |
| --- | --- | --- | --- | --- | --- |
| HLA-A*30:01 | KSKHNVTK | KREQAQCN | NFE2L3 | 38.4 | 32826.3 |
| HLA-C*03:04 | MAMGLMCGL | MAMGLMCGR | OXA1L | 12.7 | 7895.2 |
| HLA-C*03:04 | FAAFTIQQI | FAAFTIQQN | ZNF292 | 14.3 | 8266.7 |
| HLA-B*40:01 | VENDGPEPL | VENDGPEPS | PPP2R3A | 30.8 | 14443.1 |
| HLA-A*30:01 | STHILPPSI | STPYSPTQH | FAM49B | 76.8 | 24289.2 |
| HLA-B*40:01 | KEEERMYAM | KKEERMYAM | TNRC18 | 83.4 | 14138.8 |
| HLA-C*03:04 | YPYSPTQHL | YPIFSHPAS | FAM49B | 40 | 6286.7 |
| HLA-C*03:04 | FASIRTATV | FASIRTATG | APOOL | 12 | 1716.9 |
| HLA-C*03:04 | WSLYVDGEL | WSLYVDGEW | DSCAML1 | 62.8 | 2571.1 |
| DRB1_0701 | VVTYVQAENERLTAV | VVTNVQAENERLTAV | BICD1 | 48.35 | 1952.24 |
| HLA-A*30:01 | RYKGLNLTK | RYKGLNLTE | LGALS3BP | 10.5 | 297.3 |
| HLA-A*30:01 | RYKGLNLTK | RYKGLNLTE | LGALS3BP | 10.5 | 297.3 |
| HLA-A*30:01 | RSSNHNLLA | GSSNHNLLA | COG7 | 16.6 | 205.9 |
| HLA-C*03:04 | WAFIAAGFL | GAFIAAGFL | SLC35D2 | 32.4 | 288.8 |
| HLA-C*03:04 | VVVPGDSEL | VVVPGDSDL | DGKZ | 30.7 | 200.6 |
| DRB1_0701 | GFASIRTATV | GFASIRTATG | APOOL | 75.53 | 466.5 |
| DRB1_0701 | GLLGALTSTHILP | GLLGALTSTPYSP | FAM49B | 40.1 | 204.91 |
| HLA-C*03:04 | MVSPGMGHL | MGSPGMGHL | BCL9L | 65.4 | 209.3 |

**^a^** MHC affinity score of MUT peptide; **^b^** MHC affinity score of WT peptide. Immunogenic peptides are marked in red.

**Supplemental Table 15. Statistical analyses of the mutations**

| Patient ID | All screened neoepitopes in vaccine | immunogenic mutations in all screened neoepitopes | overall percentage of immunogenic mutations in all screened neoepitopes | percentage of CD8^+^ T cell responses | percentage of CD4^+^ T cell responses |
| --- | --- | --- | --- | --- | --- |
| 1 | 13 | 5 | 38.46% | Not available | Not available |
| 2 | 15 | 8 | 53.33% | 13.33% | 33.33% |
| 4 | 30 | 10 | 33.33% | Not available | Not available |
| 6 | 16 | 14 | 87.50% | 25.00% | 37.50% |
| 7 | 15 | 15 | 100% | 26.67% | 53.33% |
| 8 | 18 | 8 | 44.44% | 0% | 38.89% |
| 12 | 22 | 14 | 63.64% | 31.81% | 40.91% |
| 14 | 17 | Not available | Not available | Not available | Not available |
| 15 | 12 | 11 | 91.67% | 33.33% | 58.33% |
| 16 | 17 | 15 | 88.24% | Not available | Not available |
| 17 | 15 | 3 | 20.00% | Not available | Not available |
| 18 | 17 | Not available | Not available | Not available | Not available |
